# High Gas Permeability in Aged Superglassy Membranes with Nanosized UiO‐66−NH_2_/cPIM‐1 Network Fillers

**DOI:** 10.1002/anie.202316356

**Published:** 2023-11-30

**Authors:** Boya Qiu, Ming Yu, Jose Miguel Luque‐Alled, Shengzhe Ding, Andrew B. Foster, Peter M. Budd, Xiaolei Fan, Patricia Gorgojo

**Affiliations:** ^1^ Department of Chemical Engineering The University of Manchester Oxford Road Manchester M13 9PL UK; ^2^ Department of Chemistry The University of Manchester Oxford Road Manchester M13 9PL UK; ^3^ Department of Chemical Engineering The University of Melbourne Melbourne VIC. 3010 Australia; ^4^ Instituto de Nanociencia y Materiales de Aragón (INMA) CSIC Universidad de Zaragoza Mariano Esquillor 50018 Zaragoza Spain; ^5^ Departmento de Ingeniería Química y Tecnologías del Medio Ambiente Universidad de Zaragoza Pedro Cerbuna 12 50009 Zaragoza Spain; ^6^ Nottingham Ningbo China Beacons of Excellence Research and Innovation Institute University of Nottingham Ningbo China 211 Xingguang Road Ningbo 315100 China

**Keywords:** Aging, Gas Separation, Metal–Organic Frameworks, Polymer of Intrinsic Microporosity, Thin Film Nanocomposite Membranes

## Abstract

Superglassy membranes synthesised by polymers of intrinsic microporosity (PIMs) suffer from physical aging and show poor gas permeance over time, especially thin membranes, due to the fast rearrangement of nonequilibrium polymer chains. Herein, we constructed a novel PIM‐1 thin film nanocomposite membrane (TFN) using nanosized UiO‐66−NH_2_ (≈10 nm)/carboxylated PIM‐1 (cPIM‐1) as the composite filler. Unlike conventional fillers, which interact with the polymer only via the surface, the UiO‐66−NH_2_/cPIM‐1 forms a stable three‐dimensional (3D) network intertwining with the polymer chains, being very effective to impede chain relaxation, and thus physical aging. Nanosizing of UiO‐66−NH_2_ was achieved by regulating the nucleation kinetics using carbon quantum dots (CQD) during the synthesis. This led to increased surface area, and hence more functional groups to bond with cPIM‐1 (via hydrogen bonding between −NH_2_ and −COOH groups), which also improved interfacial compatibility between the 3D network and polymer chains avoiding defect formation. As a result, the novel TFN showed significantly improved performance in gas separation along with reduced aging (i.e. ≈6 % loss in CO_2_ permeability over 63 days); the aged membranes had a CO_2_ permeance of 2504 GPU and ideal selectivity values of 37.2 and 23.8 for CO_2_/N_2_ and CO_2_/CH_4_, respectively.

## Graphical abstract

A novel network filler, composed of nanosized UiO‐66−NH_2_ (≈10 nm) and carboxylated PIM‐1 (cPIM‐1) was prepared and was introduced into PIM‐1 thin film nanocomposite membranes (TFN). Unlike conventional fillers, which interact with the polymer only via the surface, the UiO‐66−NH_2_/cPIM‐1 forms a stable three‐dimensional (3D) network intertwining with the polymer chains, being very effective to impede chain relaxation and physical aging.

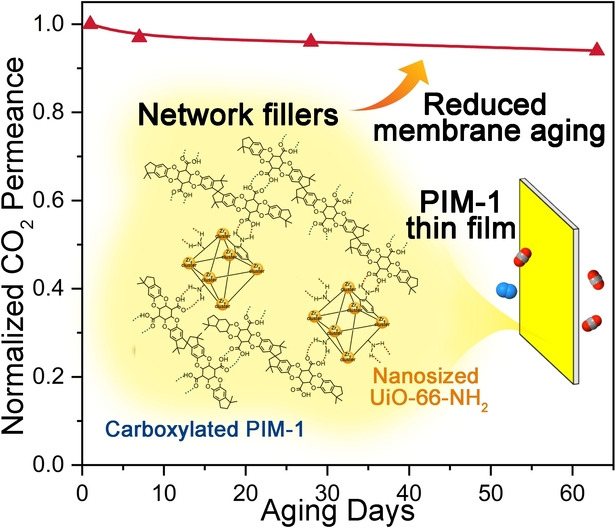

## Introduction

Compared to other technologies, membrane‐based separations possess several advantages, which include high efficiency, simple process/equipment, and low energy consumption.^[1]^ It is a promising technology for CO_2_ separation in natural gas and syngas purification and flue gas recycling. Polymers of intrinsic microporosity (PIMs) are a subclass of microporous polymers with a rigid, contorted backbone structure, providing high free volume. Compared to rubbery or inorganic membranes as well as other conventional glassy polymers (such as polyamides), PIM membranes show much higher intrinsic permeability for gases, e.g. ≈3,000 barrer for CO_2_.^[2]^ However, superglassy PIMs tend to densify over time due to the relaxation of the nonequilibrium polymer chains toward the equilibrium state, that is, physical aging. This leads to a decrease in permeability and hinders practical applications. Additionally, the selectivity of such membranes is relatively low due to the broad distribution of cavity sizes in PIMs.

Thin film composite membranes (TFCs) have an active layer thickness of <5 μm and thus tens to hundreds of times higher permeance than that of free‐standing membranes. Also, TFCs can be processed easily with good durability, being promising for practical adoptions. However, compared to thick self‐standing ones, TFCs of the most widely used PIM, PIM‐1, suffer from more rapid aging (40–90 % loss in CO_2_ permeability in just a week) due to the fast rearrangement of polymer chains within the thin active layer.^[3]^ Although the free volume of the aged PIM‐1 TFCs could be restored to a certain extent (e.g. by the exposure of the aged membranes to methanol vapor),^[4]^ intrinsic anti‐aging properties are still desired.

To reduce the aging issue, the rearrangement of the polymer chains should be constrained within the chain network of the PIM‐1 membranes. Many strategies were developed, including modification of the intrinsic topology of PIM‐1[^3^, ^5^] and preparation of PIM‐1 thin film nanocomposite membranes (TFN) with porous and non‐porous fillers. PIM‐1 structural modifications can help to reduce aging, but it is challenging to maintain separation performance at the same time.[^3^, ^5^] In addition, the modification may affect the membrane‐forming properties, increasing the brittleness of the resulting films and decreasing the reproducibility of membrane fabrication.^[6]^ Regarding the second strategy, fillers that have high sieving effects and/or CO_2_ philicity can improve the selectivity of PIM membranes as well. Yet, it is worth noting that common porous fillers, such as hypercrosslinked polystyrene (HCP)^[7]^ and silica nanosheets (SN)^[8]^ do not show good interaction with the polymer, and thus are not very effective in impeding aging. In addition, the inclusion of fillers that have low compatibility with the polymer is prone to introducing interfacial defects, which decrease the selectivity of membranes, especially for thin membranes. It has been reported that functionalized fillers, such as sulfonated (S−)SN,^[8]^ carbonized (C−)HCP,^[7]^ functionalized metal–organic frameworks (MOFs, such as UiO‐66−NH_2_),^[9]^ and porous aromatic framework (PAF),^[10]^ can interact with the PIM‐1 chains to reduce chain relaxation, and thus aging.[^7^, ^8^] In particular, PAF allows partial intrusion of polymer segments into its pores and effectively inhibits the aging in thick film PIM‐1 membranes (thickness >100 μm),^[10]^ but poorly performed in TFCs for preventing the aging.^[11]^ It is worth noting that the chain‐filler interaction only takes place on the outer surface of the fillers, and hence, for common fillers of >50 nm in size, the effective surface area is limited, making them not very ideal for reducing aging.

Here, nanosized UiO‐66−NH_2_/carboxylated PIM‐1 (cPIM‐1) was synthesised and used as a composite filler in PIM‐1‐based TFNs, forming a stable three‐dimensional (3D) network in the membrane. The network intertwines and entangles with the PIM‐1 chains, hindering chain relaxation and preventing TFN aging effectively. UiO‐66−NH_2_ was selected as the candidate for preparing the composite filler due to the predefined pore size, good stability, and high CO_2_ selectivity,^[9]^ as well as its capability to bond with cPIM‐1. The UiO‐66−NH_2_ was nanosized to ≈10 nm by regulating the nucleation kinetics using carbon quantum dots (CQD) during the synthesis. The nanosized UiO‐66−NH_2_ particles have several times more surface functional groups (−NH_2_) to bond to cPIM‐1 chains (−COOH) via hydrogen bonding than conventional large particles (≈100 nm), enabling enhanced interactions with cPIM‐1. The free carboxyl groups on the cPIM‐1 (those that are not bonded to the amine group of the MOF) can also undergo dipole‐dipole attraction to each other, leading to a stable 3D network. As a result, the novel PIM‐1 TFNs show minimal aging in CO_2_ separation (i.e. ≈6 % loss in CO_2_ permeability over 63 days). In addition, the improved interfacial compatibility between the network and polymer chains minimised the structural defects, which enhanced its ideal selectivity to 37.2 and 23.8, respectively, for CO_2_/N_2_ and CO_2_/CH_4_ systems.

## Results and Discussion

**Figure 1:**
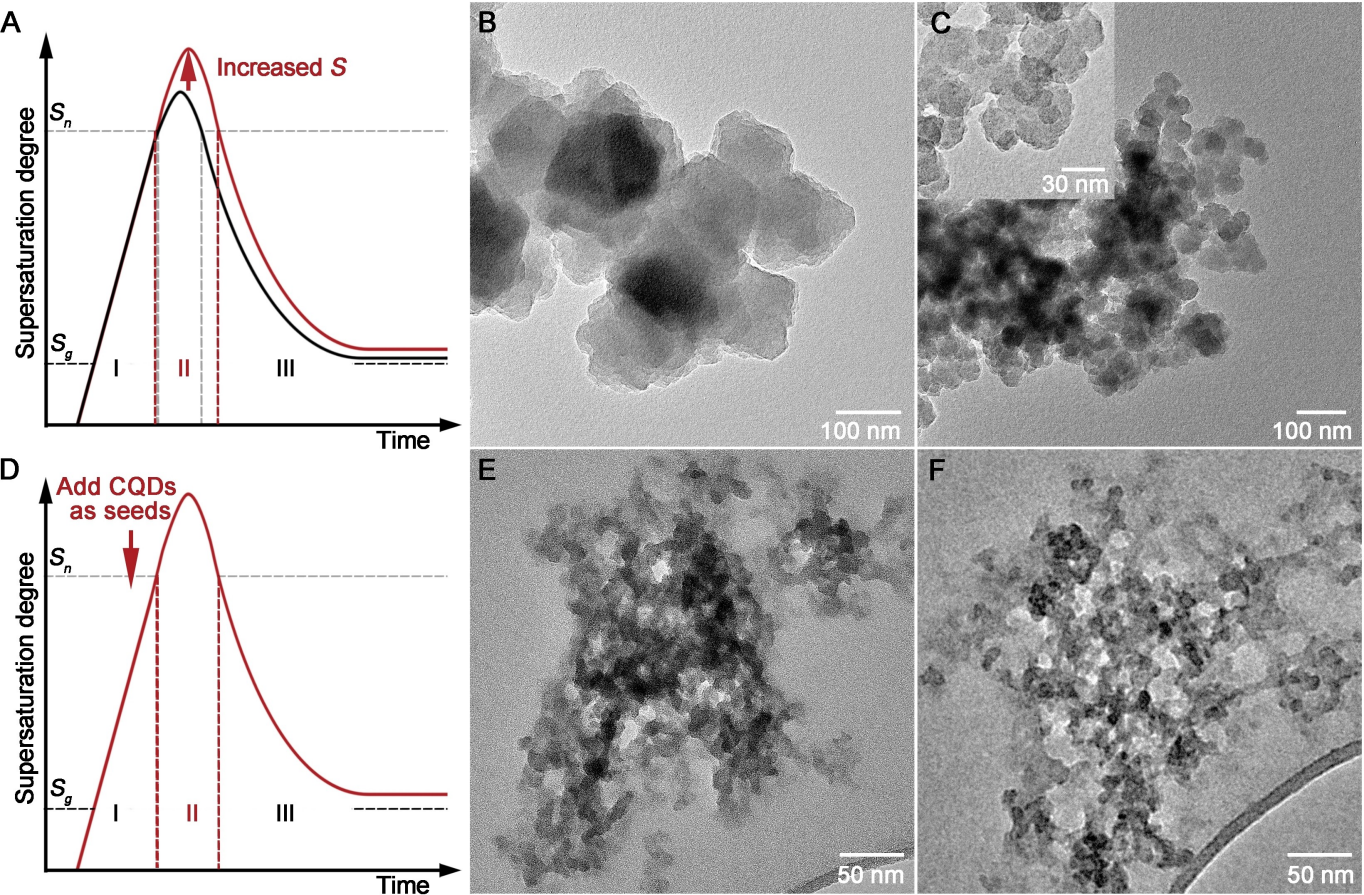
Schematics of the change of supersaturation degree as a function of time during crystallisation according to LaMer′s model (A&D, *S*_g_: critical supersaturation degree for growth); TEM morphology of L−UiO‐66−NH_2_ (B), S−UiO‐66−NH_2_ (C and the inset), C−UiO‐66−NH_2_ (E), and C−UiO‐66 (F).

We began by nanosizing the UiO‐66−NH_2_ MOF. Based on LaMer's model, crystallisation includes nucleation and crystal growth. The primary nucleation starts when the supersaturation degree surpasses the critical supersaturation degree for nucleation (*S_n_*), then consumes metal ions and ligands, and finishes when the supersaturation degree decreases to *S_n_* (Figure 1A).^[12]^ Increasing the supersaturation degree can boost nucleation and increase the number of nuclei, thus decreasing the size of the resulting MOF particles. As shown in Figure 1B–C, adding deionised (DI) water into the *N*,*N*‐dimethylformamide (DMF) mother solution was able to decrease the size of UiO‐66−NH_2_ from ≈100 nm to ≈40 nm (denoted as L−UiO‐66−NH_2_ and S−UiO‐66−NH_2_, respectively), as it led to a higher supersaturation degree by reducing the association effects between the 2‐aminoterephthalic acid (NH_2_‐BDC) molecules and accelerating their deprotonation rate.[^9b^, ^13^] However, the primary nucleation makes the system heterogeneous, leading to secondary nucleation in the presence of parent crystals due to the low energy barrier of heterogeneous nucleation, which results in polydispersed crystals (Figure 1C).

Our approach is to seed the supersaturated solution using ultrasmall heterogeneous nucleation sites (i.e. CQDs) to encourage heterogeneous nucleation instead of primary nucleation. The introduction of sufficient nucleation sites can reduce the supersaturation degree rapidly below *S_n_*, thus preventing secondary nucleation (Figure 1D). The CQDs (with average diameters of ≈2.7 nm, Figure S1A–B) possess surface carboxyl and hydroxyl groups (Figure S1C–D), which are able to bond Zr and NH_2_‐BDC via chelation and hydrogen bonding, respectively, thus making CQDs favourable candidates as the heterogeneous sites for promoting nucleation.^[14]^ Facilitated by the CQDs (as the nucleation sites), monodispersed and ultrasmall UiO‐66−NH_2_ crystals (denoted as C−UiO‐66−NH_2_) were prepared (Figure 1E and Figure S2). Especially, the ≈10 nm size of C−UiO‐66−NH_2_ is the lowest recorded in the literature for the UiO‐66 MOF.^[9]^ The strategy is generic, which was also used for the synthesis of the nanosized UiO‐66 (i.e. C−UiO‐66) with similar diameters of ≈10 nm (Figure 1F). Thanks to the ultrasmall particle size of CQDs, they do not interfere with crystallisation, and relevant properties of C−UiO‐66−NH_2_ and C−UiO‐66 are consistent with those of other MOFs prepared in this work (Figure S3 and S4).

**Figure 2:**
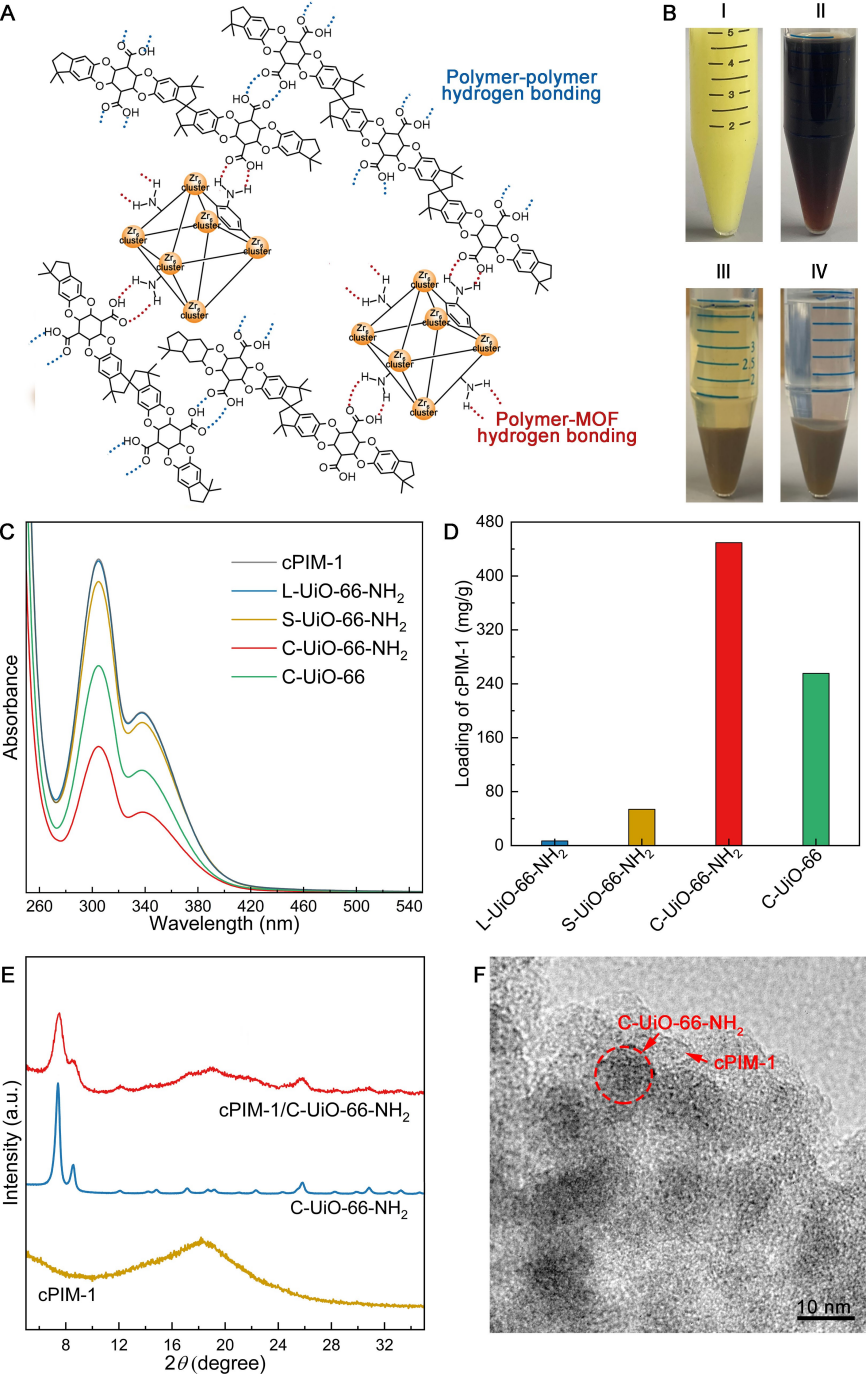
Schematic illustration of the cPIM‐1/UiO‐66−NH_2_ composite filler with the network structure (A); Photograph of the C−UiO‐66−NH_2_ (I), cPIM‐1 (II), the supernatant (unused cPIM‐1) and precipitate (cPIM‐1/C−UiO‐66−NH_2_) after mixing and centrifugation (III), and the colourless supernatant after THF washing and centrifugation (IV) (B); UV/Vis absorbance spectra of the cPIM‐1 in THF solution and the supernatant of the mixture of L−UiO‐66−NH_2_, S−UiO‐66−NH_2_, C−UiO‐66−NH_2_, or C−UiO‐66 with cPIM‐1 in THF, spectra of cPIM‐1 and the supernatant of the mixture of cPIM‐1 with L−UiO‐66−NH_2_ are too close to be differentiated visually (C); Loading of cPIM‐1 on the different MOFs in mg cPIM‐1 per gram of MOF (D); XRD spectra of cPIM‐1, C−UiO‐66−NH_2_, and cPIM‐1/C−UiO‐66−NH_2_ (E); TEM image of cPIM‐1/C−UiO‐66−NH_2_ (F).

One of the key benefits of nanosizing C−UiO‐66−NH_2_ is the increased outer surface area (based on the weight fraction), allowing greater exposure of the surface −NH_2_ groups for functionalization. Here, the UiO‐66 MOFs under investigation were functionalized by cPIM‐1 (Figure S5 and S6 and Table S1)^[15]^ by simply mixing the dispersed C−UiO‐66−NH_2_ with cPIM‐1 in tetrahydrofuran (THF). The −COOH groups of cPIM‐1 can bind to the surface −NH_2_ groups of C−UiO‐66−NH_2_ via hydrogen bonding (red dotted lines as illustrated in Figure 2A) between the two. Importantly, the remaining −COOH groups of cPIM‐1 can bond with each other via hydrogen bonding (blue dotted lines as illustrated in Figure 2A) to construct a stable cPIM‐1/C−UiO‐66−NH_2_ network (Figure 2A), and such a composite with the network structure might be able to intertwine with the PIM‐1 chain, being beneficial to retard chain relaxation, and hence membrane aging.

The integration of UiO‐66−NH_2_ and cPIM‐1 was visualized as shown in Figure 2B. The colour of the pristine C−UiO‐66−NH_2_ (in THF) and the cPIM‐1 (in THF) was pale yellow (I) and brown (II), respectively. After mixing and centrifugation, the precipitate i.e. the resulting cPIM‐1/C−UiO‐66−NH_2_ composite (III) showed a lighter brown colour, which remained unaltered after THF washing and centrifugation (IV). This and the clear supernatant in (IV) suggest a stabilized microscopic structure through hydrogen bonding (between −NH_2_ and −COOH). To estimate the cPIM‐1 loading on the MOFs, supernatants from different mixture systems were analysed by ultraviolet‐visible (UV/Vis) spectroscopy (to determine the unused cPIM‐1 contained in them), as described in the supplementary material. The cPIM‐1 (solubilized in THF) shows the characteristic absorbance band at 305 nm, and the band intensity of the supernatant decreased to different extents, as shown in Figure 2C, suggesting different loadings of cPIM‐1 for the different UiO‐66 MOFs. In detail, the intensity of the band ascribed to cPIM‐1 in the supernatant of the C−UiO‐66−NH_2_ sample mixture was the lowest, corresponding to the highest loading of cPIM‐1 on the composite (i.e. 449.4 mg/g). Conversely, the cPIM‐1 loading on S−UiO‐66−NH_2_ and on L−UiO‐66−NH_2_ was estimated as 53.8 mg/g and 6.9 mg/g, respectively (Figure 2D). The results indicate that the ultrasmall particle sizes of C−UiO‐66−NH_2_ render up to two orders of magnitude more surface functional groups available for the electrostatic interaction with cPIM‐1 than the larger UiO‐66−NH_2_. Interestingly, although C−UiO‐66 has comparable particle size to C−UiO‐66−NH_2_, its cPIM‐1 loading (via physical adsorption) was almost half (i.e. 255.5 mg/g); likewise, the loading of PIM‐1 on C−UiO‐66−NH_2_ was 243.0 mg/g (Figure S7), suggesting hydrogen bonding capabilities provided by the amine groups are important for the bonding between the cPIM‐1 and C−UiO‐66−NH_2_. Figure 2E presents the X‐ray diffraction (XRD) patterns of cPIM‐1, C−UiO‐66−NH_2_, and cPIM‐1/C−UiO‐66−NH_2_, showing the composite material (red line) with the characteristic diffraction peaks of both, the crystalline MOF phase (2*θ*=7.4°) and the amorphous hump of cPIM‐1 (2*θ*=18.2°).

Figure 2F shows the transmission electron microscope (TEM) image of cPIM‐1/C−UiO‐66−NH_2_ (cPIM‐1 loading at ≈449.4 mg/g), in which the nanosized C−UiO‐66−NH_2_ particles are found to be embedded in the cPIM‐1. The soft and non‐defective outer layer around the MOF particles attributed to hydrogen bonding is likely to avoid particle aggregation and defects in the membranes. In addition, available −COOH groups on cPIM‐1 chains that have not bonded to amine groups of the MOFs can form hydrogen bonding between them, leading to a chain network. Such a unique structure of the cPIM‐1/C−UiO‐66−NH_2_ composite filler, when dispersed into PIM‐1 thin films, could prevent the aging issue and improve gas separation. In particular, the network structure could intertwine and entangle with PIM‐1 chains to hinder chain relaxation, and the structural similarity between PIM‐1 and cPIM‐1 could avoid the formation of interfacial defects between the filler and polymer chains, which is crucial to obtain good selectivity properties.

The PIM‐1 TFNs were prepared using the composite filler with the loading at 5–10 wt. % (Figure S8).

**Figure 3:**
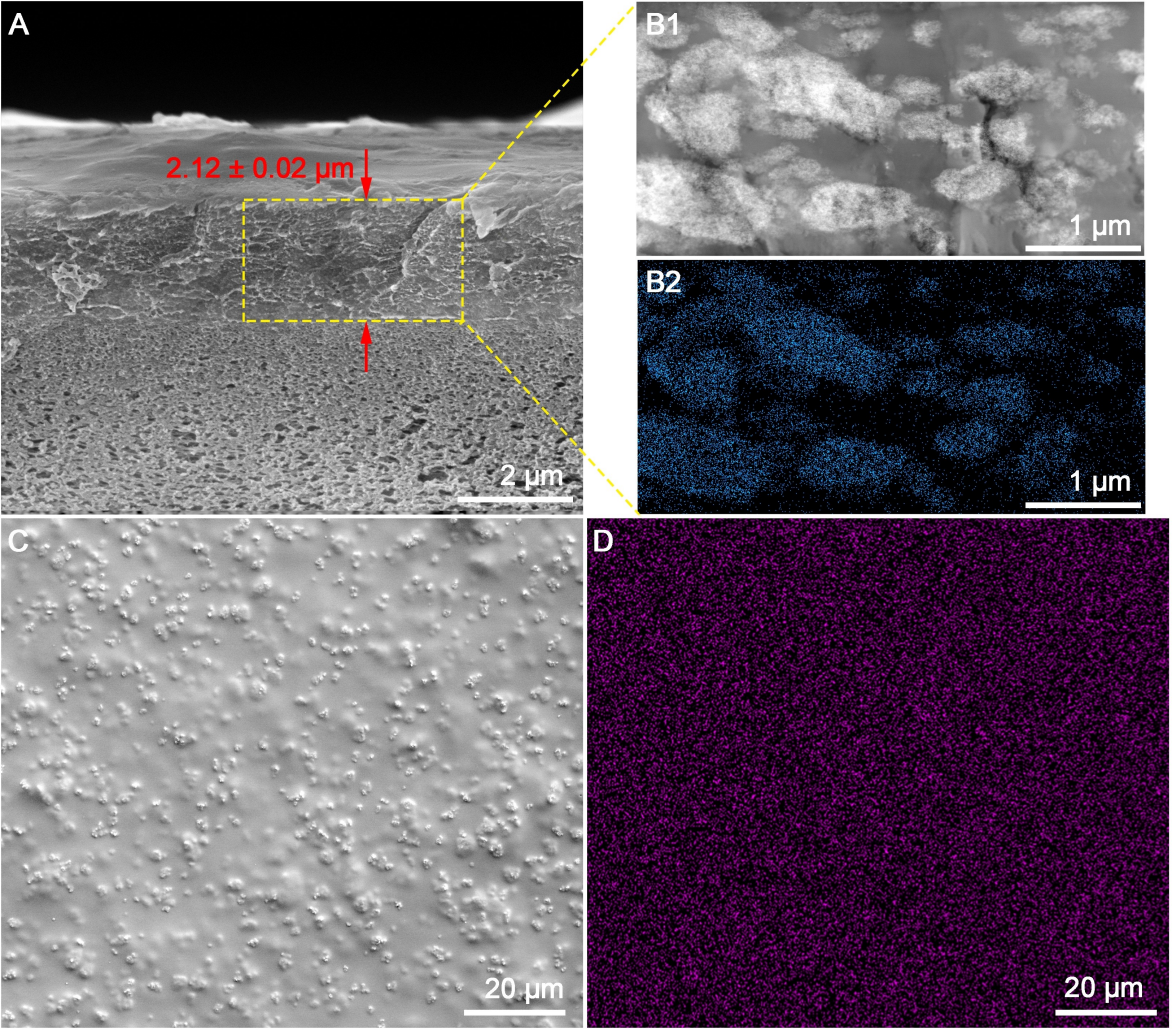
Morphology of the PIM‐1 TFN with 7.5 wt. % cPIM‐1/C−UiO‐66−NH_2_ as the filler: SEM of the cross‐section of the TFN (A); HADDF‐STEM (B1) and EDS mapping for Zr (B2) of the cross‐section slides of the TFN; SEM (C) and EDS mapping for Zr (D) of the membrane surface.

The pristine PIM‐1 TFC and PIM‐1 TFNs with fillers have comparable active layer thickness of ≈2 μm (Figure S9). Exemplified by the PIM‐1 TFN with 7.5 wt. % cPIM‐1/C−UiO‐66−NH_2_ loading, the cross‐sectional scanning electron microscopy (SEM) micrograph (Figure 3A) shows a thickness of 2.12±0.02 μm). Based on the high‐angle annular dark‐field scanning transmission electron microscopy image (HADDF‐STEM) of the membrane cross‐section (Figure 3B1), the fillers in the PIM‐1 matrix showed network structures. Energy dispersive X‐ray spectroscopy (EDS) mapping of Zr confirms the location of the network fillers and suggests an even distribution of the C−UiO‐66−NH_2_ in the network fillers (Figure 3B2). For the PIM‐1 TFN with 7.5 wt. % of cPIM‐1/C−UiO‐66−NH_2_, the percentage area covered by the network fillers is approximately 70 %. This high coverage potentially ensured effective confinement of the PIM‐1 chains in the polymer matrix and prevented membrane aging. Besides, the even distribution of the network filler throughout the intramembrane space was also proved by the even distribution of Zr in EDS mapping of the membrane surface (Figure 3C–D).

**Figure 4:**
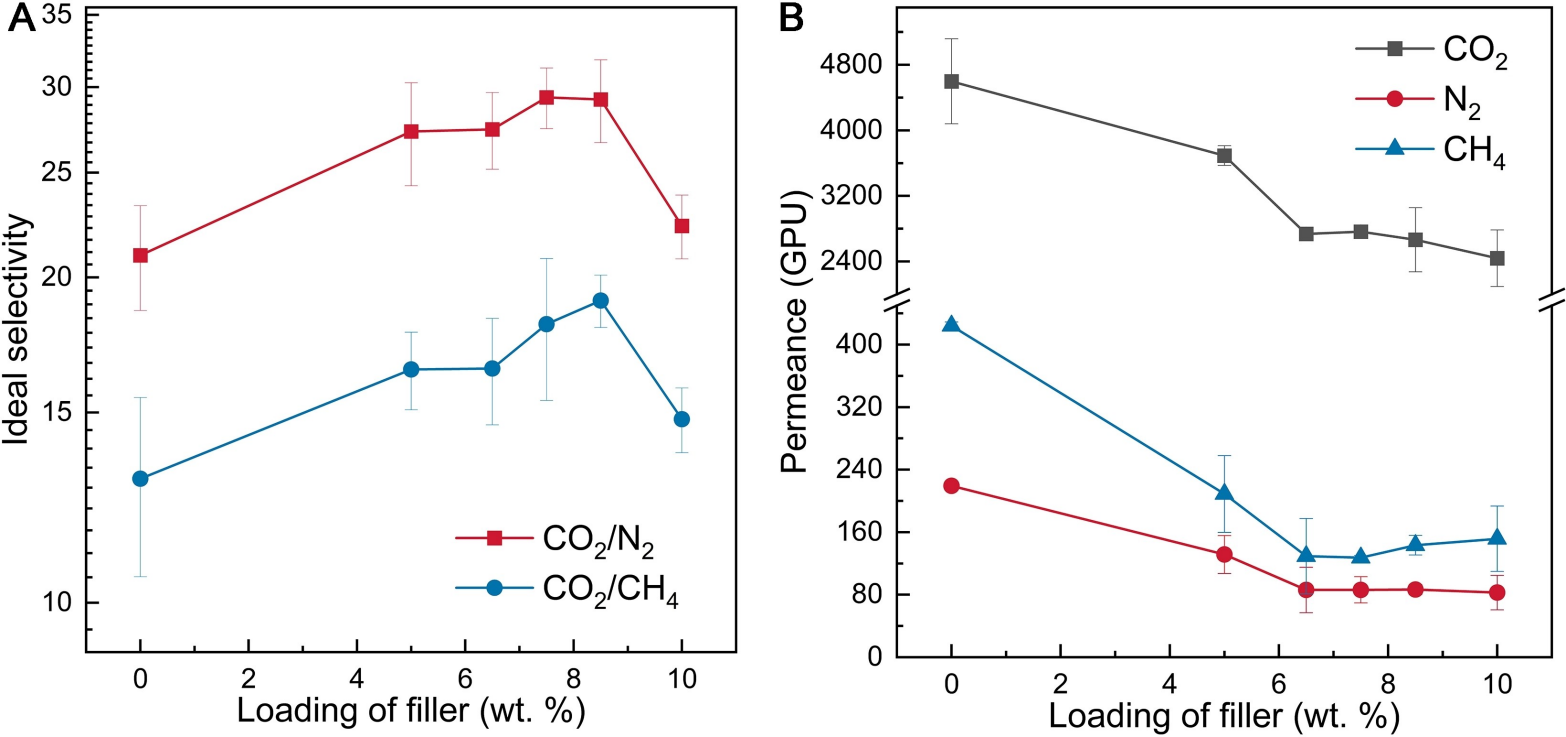
Separation performance of the PIM‐1 TFN with the cPIM‐1/C−UiO‐66−NH_2_ composite filler at different loadings: ideal CO_2_/N_2_ and CO_2_/CH_4_ selectivity (A); Gas permeance (B). (Permeance and selectivity are calculated based on the average of at least three measurements)

The PIM‐1 TFNs were assessed by single gas permeation using CO_2_, N_2_, and CH_4_ and ideal selectivity values were estimated. Figure 4A shows that the ideal CO_2_/N_2_ and CO_2_/CH_4_ selectivity increases up to maximum values of ≈29 and ≈19 for CO_2_/N_2_ and CO_2_/CH_4_, respectively, for filler loadings in the range 7.5–8.5 wt. %. These are 40 % and 46 % higher than those of the pristine PIM‐1 TFCs (≈21 for CO_2_/N_2_ and ≈13 for CO_2_/CH_4_). The initial enhancement in the selectivity could be attributed to (i) selective separation via C−UiO‐66−NH_2_ ^[15]^ and (ii) suppressed interfacial defects (between the PIM‐1 phase and the composite filler phase).^[7]^ Excessive loading of the composite filler (beyond 8.5 wt. %) may lead to ineffective attachment of the PIM‐1 polymer chains to the cPIM‐1‐MOF 3D structure, producing larger regions of unaltered PIM‐1 which jeopardise the selectivity. The PIM‐1 TFNs have good reproducibility, proved by the relatively low error bars in the selectivity and the permeance (Figure 4).

It is worth noting that filler inclusion in the PIM‐1 TFNs compromised the gas permeance of the resulting TFNs (Figure 4B), possibly due to polymer rigidification arising from the interaction between the filler and the polymer,^[16]^ and the intrinsic lower permeability of the filler as compared to PIM‐1. For example, the CO_2_ permeance of PIM‐1 TFN with 7.5 wt. % cPIM‐1/C−UiO‐66−NH_2_ was 2763 GPU, which is much lower than that of the pristine PIM‐1 thin film membranes (4599 GPU). Therefore, considering the trade‐off between membrane permeability and selectivity, the PIM‐1 TFNs with 7.5–8.5 wt. % cPIM‐1/C−UiO‐66−NH_2_ exhibited the best overall performance among the membranes investigated here, as well as the state‐of‐the‐art (Figure S10). Also, the best TFN in this work demonstrated better performance in a binary CO_2_/N_2_ separation system than the pristine PIM‐1 TFC (i.e. the initial membrane selectivity: 13.3 for the PIM‐1 TFC vs. 20.5 for the PIM‐1 TFN with 8.5 wt. % cPIM‐1/C−UiO‐66−NH_2_, Table S2).

**Figure 5:**
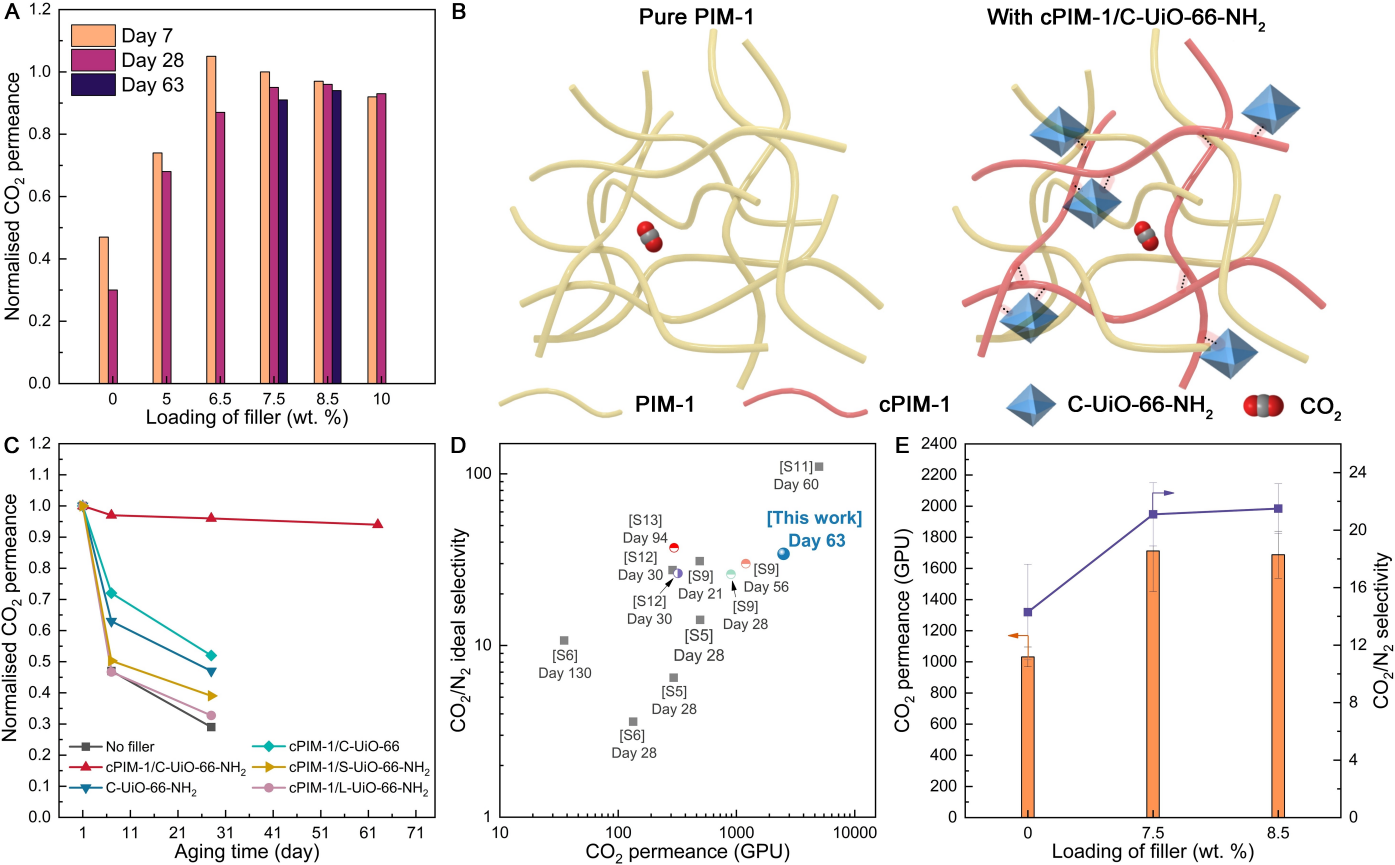
Normalised CO_2_ permeance of PIM‐1 TFC and PIM‐1 TFNs with different cPIM‐1/C−UiO‐66−NH_2_ loadings (A); Schematic illustration of the cPIM‐1/UiO‐66−NH_2_ composite filler entangling with PIM‐1 chains for preventing chain relaxation (B); Comparison of the normalised CO_2_ permeance for the best performing PIM‐1 TFN containing 8.5 wt. % of cPIM‐1/C−UiO‐66−NH_2_ with the relevant control membranes (pure PIM‐1 TFCs and PIM‐1 TFNs containing 8.5 wt. % of cPIM‐1/C−UiO‐66, C−UiO‐66−NH_2_, cPIM‐1/S−UiO‐66−NH_2_, or cPIM‐1/L−UiO‐66−NH_2_) (C); Ideal CO_2_/N_2_ selectivity vs. gas permeance plots for the aged PIM‐1 TFCs and TFNs (grey squares refer to relevant PIM‐1 TFCs, circles refer to PIM‐1 TFNs with different fillers (See Supporting Information for full data set, Table S4)) (D); Mixed gas separation performance of the PIM‐1 TFC and optimised PIM‐1 TFNs after 7‐day aging (E).

In addition to the improved selectivity, the inclusion of the cPIM‐1/C−UiO‐66−NH_2_ composite filler in the PIM‐1 TFN significantly reduced the physical aging. Figure 5A shows the comparison of the normalised CO_2_ permeance of the TFNs with different filler loadings on Day 7 and 28, and for the best performing ones also on Day 63 (using all Day 1 CO_2_ permeance as the reference value for the calculations), the absolute permeance and selectivity data are presented in Table S3. The pristine PIM‐1 TFC showed the lowest normalised CO_2_ permeance (0.29), thus the most significant aging, viz. ≈71 % loss in CO_2_ permeance after 28 days. Again, the PIM‐1 TFN with 7.5–8.5 wt. % loading of cPIM‐1/C−UiO‐66−NH_2_ showed the comparatively best anti‐aging performance, i.e. for that with 8.5 wt. % filler, the CO_2_ permeance only decreased by approximately 4 % and 6 % after 28 and 63 days, respectively.

The improved membrane performance of the developed TFNs could be attributed to the 3D network formed by the cPIM‐1/C−UiO‐66−NH_2_ composite filler, which runs throughout the entire PIM‐1 matrix (as illustrated by Figure 5B). The 3D network could interlace and intertwine with the PIM‐1 matrix, thus providing a much stronger restriction to the rearrangement of the PIM‐1 chains compared to other fillers. One key to the network is the intermolecular hydrogen bonding between respective −COOH groups on cPIM‐1 and −NH_2_ groups on the C−UiO‐66−NH_2_. To test the hypothesis above, we prepared relevant control TFNs with the composite filler of cPIM‐1/C−UiO‐66 (without −NH_2_ group) and C−UiO‐66−NH_2_ (without introducing cPIM‐1 and thus no −COOH groups). As shown in Figure 5C (with similar filler loading amounts of ≈8.5 wt. %), the normalised CO_2_ permeance of cPIM‐1/C−UiO‐66 TFN (without the −NH_2_ group) after 7 and 28 days was 0.63 and 0.47, respectively. The fast aging could be attributed to the loose and unstable network of the filler, since the loading of cPIM‐1 on C−UiO‐66 is low (Figure 2C–D), and the interaction between them could be weak. Likewise, without introducing the −COOH group, the network formation could be hindered, and the normalised CO_2_ permeance for C−UiO‐66−NH_2_ TFN (without the −COOH group) after 7 and 28 days was 0.72 and 0.52, respectively (Figure 5C).

Another key to the network is the hydrogen bonding between the −COOH groups on the cPIM‐1/C−UiO‐66−NH_2_ composites, and thus, substantial cPIM‐1 loading in filler is necessary. To validate the hypothesis above, control TFNs were prepared with the composite filler of cPIM‐1/L−UiO‐66−NH_2_ and cPIM‐1/S−UiO‐66−NH_2_ (with low cPIM‐1 loading in the filler of 6.9 and 53.5 mg/g, respectively) for comparison. Both the L−UiO‐66−NH_2_ and S−UiO‐66−NH_2_ TFNs show fast aging (with the normalised CO_2_ permeance of 0.33 and 0.39, respectively, after 28 days, Figure 5C). Membranes from the four control experiments showed a significant decrease in CO_2_ permeance over time, proving the critical role played by the hydrogen bonding and the resulting 3D network for preventing aging.

By comparing with the state‐of‐the‐art (Table S4), the PIM‐1 TFN with the cPIM‐1/C−UiO‐66−NH_2_ composite filler shows one of the lowest decreases in CO_2_ permeance over time (6 % in 63 days). In detail, after 63 days of aging, the PIM‐1 TFN (with 8.5 wt. % composite filler) maintained a CO_2_ permeance of 2504 GPU and showed an ideal CO_2_/N_2_ and CO_2_/CH_4_ selectivity of 37.2 and 23.8, respectively, which is also among the best performances after aging in comparison with the published data (Figure 5D and Figure S11).

Previously, Yu et al.^[6]^ developed branched cPIM‐1 TFCs with higher CO_2_ permeance at ≈3200 GPU and higher ideal CO_2_/N_2_ selectivity at 50–90 (Figure 5D). However, in a real CO_2_/N_2_ binary system, the CO_2_ permeance of branched cPIM‐1 TFC was low (at 700–1000 GPU) with poor selectivity of 13–19, which was lower than the non‐carboxylated branched PIM‐1 counterpart (CO_2_/N_2_=16–19).^[17]^ Comparatively, using the linear PIM‐1, the PIM‐1 TFNs developed in our work containing 7.5–8.5 wt. % C−UiO‐66−NH_2_/cPIM‐1 showed the higher CO_2_ permeance (≈1700 GPU) and CO_2_/N_2_ selectivity (≈21) for the real CO_2_/N_2_ binary system after aging (Table S2), representing the best reported performance in the literature. The 30–40 % reduction in CO_2_ permeance and ≈30 % in selectivity compared to the ideal results may be due to CO_2_‐induced plasticization and the competition for sorption sites between CO_2_ and N_2._ ^[18]^

Importantly, the CO_2_ permeability and CO_2_/N_2_ selectivity of the 7‐day aged optimised PIM‐1 TFN were ≈65 % and ≈50 % higher than that of the aged PIM‐1 TFC, respectively (Figure 5E), demonstrating the significant role of the novel cPIM‐1/C−UiO‐66−NH_2_ composite filler in retarding aging.

## Conclusion

In this study, UiO‐66−NH_2_ was successfully nanosized to ≈10 nm for the first time by regulating the nucleation kinetics using CQD. In detail, the CQD provided sufficient heterogeneous nucleation sites before primary nucleation occurred, thus increasing the number of nuclei, rapidly decreasing the supersaturation degree of reactants, and avoiding secondary nucleation. The nanosized UiO‐66−NH_2_ particles exposed more surface −NH_2_ groups to bond −COOH groups of cPIM‐1 by hydrogen bonding. This allowed the formation of a stable 3D network which was used as filler for the preparation of thin films of PIM‐1.

In the thin layer of the developed PIM‐1 TFN (≈2 μm thick), the 3D network intertwined with PIM‐1 chains across the whole membrane network, preventing chain relaxation significantly and thus physical aging. For CO_2_ separation from single gas measurements, there was only ≈6 % loss in permeability over 63 days.

Additionally, the interfacial defects were diminished due to the hydrogen bonding between nanosized UiO‐66−NH_2_ and cPIM‐1 and the tight entanglement between the cPIM‐1 and PIM‐1. The novel TFN also showed improved selectivity to CO_2_ separation from the model CO_2_/N_2_ and CO_2_/CH_4_ binary systems with ideal selectivities of 37.2 and 23.8, respectively. This was due to the sieving effect of the nanosized UiO‐66‐NH_2_.

This work provides a generic solution to address the physical aging of PIM‐1 membranes, which is one of the major issues preventing their use in industrial applications. In addition, due to the wide range of hydrogen bonds and the diversity of MOFs, the 3D network can be synthesised in diverse ways and has great potential to be applied in various glassy polymers to prevent aging and improve the target selectivity.

## Conflict of interest

The authors declare no conflict of interest.

1

## Supporting information

As a service to our authors and readers, this journal provides supporting information supplied by the authors. Such materials are peer reviewed and may be re‐organized for online delivery, but are not copy‐edited or typeset. Technical support issues arising from supporting information (other than missing files) should be addressed to the authors.

Supporting Information

## Data Availability

The data that support the findings of this study are available from the corresponding author upon reasonable request.
