# Supplementary material for: High Gas Permeability in Aged Superglassy Membranes with Nanosized UiO‐66−NH2/cPIM‐1 Network Fillers
Source: Angew Chem Int Ed Engl. 2023 Nov 30;63(1):e202316356. doi: 10.1002/anie.202316356 (PMC10952568; doi:10.1002/anie.202316356)
Supplement: Supplementary file 1 — Supporting Information [file ANIE-63-0-s001.pdf]

Angewandte Chemie
Eine Zeitschrift der Gesellschaft Deutscher Chemiker

Supporting Information

High Gas Permeability in Aged Superglassy Membranes with Nanosized UiO-66–NH_2/cPIM-1 Network Fillers

B. Qiu, M. Yu, J. M. Luque-Alled, S. Ding, A. B. Foster, P. M. Budd, X. Fan, P. Gorgojo**

Supporting Information for

High Gas Permeability in Aged Superglassy Membranes with Nanosized UiO-66-NH_2/cPIM-1 Network Fillers

Boya Qiu[1], Ming Yu[2,3], Jose Miguel Luque-Alled[4,5], Shengzhe Ding[1], Andrew B. Foster[2], Peter M. Budd[2], Xiaolei Fan[1,6*], Patricia Gorgojo[1,4,5*]

[1]*Department of Chemical Engineering, The University of Manchester, Oxford Road, Manchester M13 9PL, United Kingdom*

[2]*Department of Chemistry, The University of Manchester, Oxford Road, Manchester M13 9PL, United Kingdom*

[3]*Department of Chemical Engineering, The University of Melbourne, Melbourne, VIC. 3010, Australia*

[4]*Nanoscience and Materials Institute of Aragón (INMA) CSIC-Universidad de Zaragoza, Mariano Esquillor, 50018 Zaragoza, Spain*

[5]*Chemical and Environmental Engineering Department, Universidad de Zaragoza, Pedro Cerbuna 12, 50009 Zaragoza, Spain*

[6]*Nottingham Ningbo China Beacons of Excellence Research and Innovation Institute, University of Nottingham Ningbo China, 211 Xingguang Road, Ningbo 315100, China*

[*] Correspondence authors' emails: xiaolei.fan@manchester.ac.uk (X.F.); pgorgojo@unizar.es (P.G.)

Methods

Synthesis of polymers

PIM-1 was synthesized using a high-temperature method [1] with the modified solvent system. In detail, 17.03 g (50 mmol) 5,5',6,6'-tetrahydroxy-3,3,3',3'-tetramethyl-1,1'-spirobisindane (Alfa Aesar, 97%), 9.99 g (50 mmol) tetrafluoroterephthalonitrile (Fluorochem, >99%) and 20.73 g (150 mmol) potassium carbonate (K_2CO_3, Fisher Scientific, anhydrous, ≥99.5%) were added into a 500 mL three-neck round-bottom flask. The flask was connected to an N_2 purge inlet, an open-end coil condenser, and an overhead stirrer (Hei-TORQUE Expert 100, Germany). A Heating block (Asynt, UK) was used to fit the round bottom flask and placed on top of a hot plate magnetic stirrer (IKA, UK) with the temperature set at 160 °C. Dimethylacetamide (DMAc, Sigma-Aldrich, anhydrous, 99.8%), and toluene (Sigma-Aldrich, ACS reagent, ≥99.7%) were mixed at a ratio of 2:1 as the solvent of polymerization. N_2 flow was turned on to purge the system and kept open throughout the reaction. To start the reaction, 180 mL solvent was added into the flask. Heat and overhead stirrer (at 250 rpm) were then turned on immediately. The stir speed was increased periodically to provide a better mixing as polymerization resulted in increasing viscosity over time. After 30 min, polymerization was quenched with excess methanol (Sigma-Aldrich, ACS reagent, ≥99.8%). PIM-1 precipitation was collected via vacuum filtration and re-dissolved in 550 mL chloroform. Then PIM-1 was precipitated by pouring the solution into methanol (2 L) and vacuum filtered again. PIM-1 was refluxed in deionised (DI) water overnight and

vacuum filtered again. PIM-1 was immersed in a minimum amount of 1,4-dioxane (Sigma-Aldrich, anhydrous, 99.8%) for 15 min and washed by copious amounts of acetone (Sigma-Aldrich, for analysis) and methanol, and then immersed in methanol overnight. Finally, vacuum filtered PIM-1 was dried at 100 ℃ oven for one day followed with 120 ℃ vacuum oven for another two days.

Carboxylated PIM-1 (cPIM-1) was synthesised via a modified acid hydrolysis method [2]. In detail, 0.6 g of PIM-1, 36 mL of DI water, 12 mL of glacial acetic acid (Fluorochem Limited, ≥99%), and 36 mL of sulfuric acid (Thermo Fisher Scientific, ≥95%) were added sequentially to a 250 mL round-bottom flask, and a water condenser was attached. The solid-state acid hydrolysis reaction was stirred at 300 rpm for 48 h at 150 °C. Upon cooling, the heterogeneous solution was neutralised in a beaker with 100 mL of DI water and the brown powder was filtered. To remove residual reagents, the powder was refluxed in a slightly acidic DI water solution with 200 mL of DI water and 3~4 drops of sulfuric acid for approximately 12 h, filtered, and vacuum-dried at 130 °C overnight.

Synthesis of carbon quantum dots (CQDs)

1 g citric acid (Scientific Laboratory Supplies, ≥99%) solid powder was put into a glass beaker covered with a glass slide and was heated at 180 ℃ for 150 min under air. After the reaction, a yellow powder containing CQDs was produced, and the CQDs were then dispersed in water by stirring for 10 min, followed by neutralization with 5.0 M sodium hydroxide (NaOH, Sigma-Aldrich, 97%) solution to pH=7. The resultant CQDs

solution was dialyzed using Slide-A-Lyzer G2 Dialysis Casette (2K MWCO, Thermo Fisher Scientific) and the dialysis process was repeated for ~4 weeks until there was no significant change of conductivity of the surrounding distilled water.

Synthesis of C-UiO-66-NH_2 and control MOFs

C-UiO-66-NH_2 was synthesised via a modified solvothermal method [3]. Zirconium (IV) chloride ($ZrCl_4$, Thermo Fisher Scientific, 98%), 2-aminoterephthalic acid (Sigma-Aldrich, 99%), and *N*,*N*-dimethylformamide (DMF, Honeywell, ≥99.9%) was mixed in a 2.3 mmol:2.3 mmol:30 mL ratio. 113.5 mmol of water with (10 mg/mL) CQD was then slowly added to the slurry reaction solution, the slurry was introduced in a 100 ml Teflon-lined autoclave and heated at 120 °C for 24 h. The resulting crystals were separated by centrifugation, followed by washing with methanol twice to remove the excess of unreacted ligand before being refluxed in methanol at 90 °C overnight.

As the control samples, L-UiO-66-NH_2 and S-UiO-66-NH_2 were prepared via the same method without the adding of the DI water and CQD, respectively, in the slurry before the slurry was introduced in the Teflon-lined autoclave. C-UiO-66 was prepared via the same method with the same mole amount of terephthalic acid (Sigma-Aldrich, 98%) substituting 2-aminoterephthalic acid.

Determination of cPIM-1 loading on different MOFs

1 mg MOF was dispersed into 1 mL cPIM-1 solution in THF (0.8 mg/mL). The mixtures were vigorously stirred for 5 min and centrifuged at 10,000 rpm for 5 min (SIGMA 3-16KL IVD). The clear supernatants (THF containing residual cPIM-1) were diluted by

10 times with THF before measurement by ultraviolet-visible (UV-Vis) spectroscopy (Shimadzu UV-2600).

The loading of cPIM-1 was calculated based on Eq. (S1).

$$Loading\ of\ cPIM\text{-}1 = \frac{\left(C_0 - C_{\mathrm{supernatant}}\right)\cdot V}{m} \times 100\% \quad \text{(S1)}$$

where C_0 and $C_{\mathrm{supernatant}}$ are the concentration (mg/L) of cPIM-1 in THF and in the supernatant, respectively; V is the volume of the mixture; m is the mass of MOFs.

Thin-film (nano) composite membranes

Typically, 60 mg C-UiO-66-NH_2 was dispersed in 5 mL tetrahydrofuran (THF) and was mixed with 30 mg of cPIM-1 in 3 mL THF. After vigorous stirring for 5 min, the cPIM-1/C-UiO-66-NH_2 was separated by centrifugation, washed by THF twice and diluted into a certain concentration in THF. PIM-1 was dissolved in chloroform (9 mL, Acros Organics, ≥99.8%) and was mixed with the particle suspension in THF (1 mL) as the coating solution. The concentration of PIM-1 in the coating solution is typically 4.5 wt. %. TFN membranes were prepared with loadings of filler at 5, 6.5, 7.5, 8.5, and 10 wt. %. For the PIM-1 TFC membranes without filler, 1 mL THF was added into the PIM-1 solution in chloroform (9 mL) instead.

PIM-1 thin film composite (TFC) membranes were prepared using a roller-coater on a polyacrylonitrile (PAN, Solecta) substrate, the detail of coating procedure was reported in our previous work [4].

Gas permeation test

Single gas permeation test was conducted on a lab-made gas separation rig via the

standard variable method. The detail was reported in our previous work [4].

The membrane permeance was calculated based on Eq. (S2).

$$K = \frac{Q}{tAp} \times 10^6 \tag{S2}$$

where K is the gas permeance (GPU, 1 GPU = 10^{-6} cm^3(STP)/(cm^2 s cmHg) = 3.348×10^{-1} mol/(m^2 s Pa)); t is the permeation time (s), Q is the volume of gas permeating through the membrane during the defined time (cm^3, corrected to STP [0°C, 1 atm]), A is the active permeation area (cm^2), and p is the pressure difference between the feed side and the permeate of the membrane (cmHg), respectively. Herein, 35 psi of pressure difference was applied in the single gas permeation test.

The ideal selectivity was calculated based on Eq. (S3).

$$\alpha_{CO_2/x} = \frac{K_{CO_2}}{K_x} \tag{S3}$$

where K_{CO2} represents the permeance (GPU), and x refers to the N_2 or CH_4.

At least three membranes of each sample were tested for checking the reproducibility, and the averaged data with standard deviation are reported.

Mixed gas test was carried out using a 50% CO_2/50% N_2 mixture at room temperature and 40 psi (the upstream absolute pressure). The pressure of downstream absolute permeate was slightly higher than the atmosphere pressure, at 15 psi. A PoraPlotU gas chromatograph column (Agilent 490 microGC), equipped with a thermal conductivity detector located at the end of the column allowed quantification of gas composition.

Characterization

Proton nuclear magnetic resonance (^{1}H NMR) spectra were obtained from PIM-1 in deuterated chloroform (Sigma-Aldrich, 99.8 atom %D) or cPIM-1 in deuterated dimethyl sulfoxide (Sigma-Aldrich, 99.9 atom %D) on a Bruker Avance III instrument with an Oxford AS600 Magnet equipped with a cryoprobe [5 mm CPDCH 13C-1H/D] (600 MHz) at 298 K.

Number-average molar mass (M_n), weight-average molar mass (M_w), and dispersity ($Đ$) of the PIM-1 polymer were obtained using a gel permeation chromatography (GPC) fitted with a Viscotek VE2001 SEC solvent/sample module with two PL Mixed B columns and a Viscotek TDA 302 triple detector array (refractive index, light scattering, viscosity detectors). Sample was prepared as 1 mg/mL chloroform solution and filtered by a polytetrafluoroethylene (PTFE) membrane filter (0.45 μm, Fisherbrand) before analysis. The PIM-1 polymer exhibited M_n = 41300 g/mol, M_w = 95800 g/mol, and $Đ$ = 2.3.

The obtained materials were investigated by a Fourier transform infrared (FTIR) spectrometer (Shimadzu, IRTracer-100) equipped with an attenuated total reflectance cell in the wavenumber range of 4,000–400 cm^{-1}. The crystalline structures of fillers in this work were characterized using powder X-ray diffraction (XRD, Bruker D8 Discover GIXRD Autochanger) with a Cu Kα anode operated at 40 mA and 40 kV. Synthesized powders were crushed using a pestle and mortar. A small amount of sample was then mounted onto the sample holder and flattened using a glass slide. X-Ray

Photoelectron Spectroscopy (XPS) measurements were performed with an Axis Ultra spectrometer (Kratos Analytical Limited, Manchester, UK) using a monochromatic Al Kβ source (1486.7 eV). The fillers were drop-cast onto silicon substrates at a sufficient concentration to allow complete coverage of the surface and dried at room temperature before the analysis.

The cross-sectional and surface morphology of the prepared membranes were investigated by scanning electron microscope (SEM, FEI Quanta 650 FEG(E)SEM+EDX) operated at an accelerating voltage of 5 kV. Samples were prepared by fracturing the membranes in liquid nitrogen and were coated with a thin layer of gold/palladium (Au/Pd, 80/20) using a sputter coater (Quorum Q150R Plus - Rotary Pumped Coater). Transmission electron microscopy (TEM) was conducted with an FEI Tecnai F30 FEG-AEM TEM operating at 300 kV for the CQDs, and with FEI Talos F200A AEM operated at an accelerating voltage of 80 kV for the MOFs. High-angle annular dark-field scanning transmission electron microscopy (HAADF-STEM) images were recorded on an FEI Talos F200A AEM operated at an accelerating voltage of 300 kV, equipped with a Schottky field-emission gun (X-FEG) operating at an extraction voltage of 4.5 kV, a monochromator (energy spread ~0.25 eV) and an FEI Super-X 4-detector EDX system. For the preparation of the HAADF-STEM specimens, the TFN samples were embedded in EpoThin 2 epoxy resin overnight. The block of resin was trimmed with a glass knife (Leica EM UC7 ultramicrotome), resulting in a pyramid-like shape with a narrow window exposing the cross-section of the TFN

samples. Ultrathin sections of 500 nm were obtained using a diamond knife (Leica EM UC7 ultramicrotome) at room temperature. The angle of the knife was set at 6° and the speed at 1 mm/s. The ultrathin sections were mounted on carbon 300 mesh copper grids.

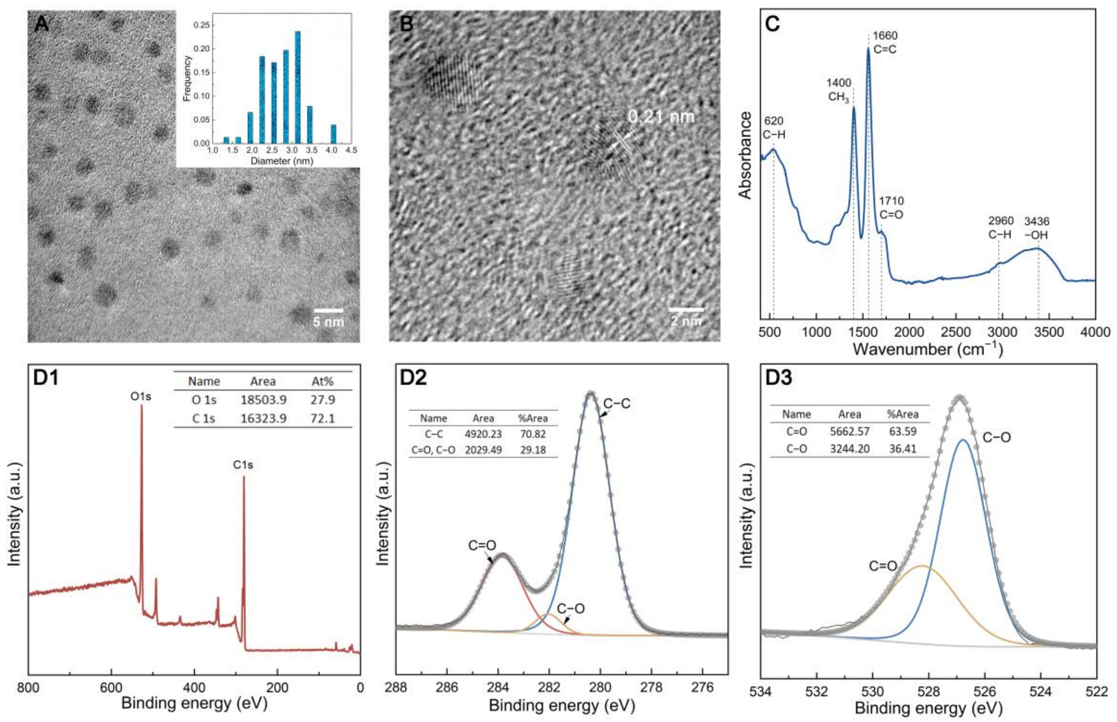

Figure S1. Characterization of carbon quantum dots (A: TEM image (inset shows the size distribution of CQD); B: Lattice parameter in high resolution transmission electron microscope (HRTEM); C: FTIR; D: XPS (survey spectrum (D1); high-resolution spectra of C1s (D2) and O1s (D3)).

Figure S1A shows the TEM images of the obtained CQDs. The CQDs are well dispersed with the average diameter being 2.7 nm (**Figure S1A inset**). The *d*-spacing of CQD obtained from HRTEM is approximately 0.21 nm (**Figure S1B**). The functional groups were further detected by FTIR, as shown in **Figure S1C**, the stretching vibrations of C−OH (3436 cm^{-1}), C−H (2960 and 2870 cm^{-1}), C=O (1710 cm^{-1}), and C=C (1660 cm^{-1}) were observed. The elemental composition and carbon bonding configurations of the CQDs were quantified by XPS measurements (**Figure S1D**). In the high-resolution C1s spectra, the peaks at binding energies of 280.8, 282.5, and 284.2 eV are attributed to sp^2 carbon atoms, alcoholic (C−OH) and carbonyl (C=O) carbon atoms, respectively. In accordance with it, peaks of C−OH and C=O (at binding energies of 536.8 and 528.2 eV, respectively) were also shown in the high-resolution O1 spectra.

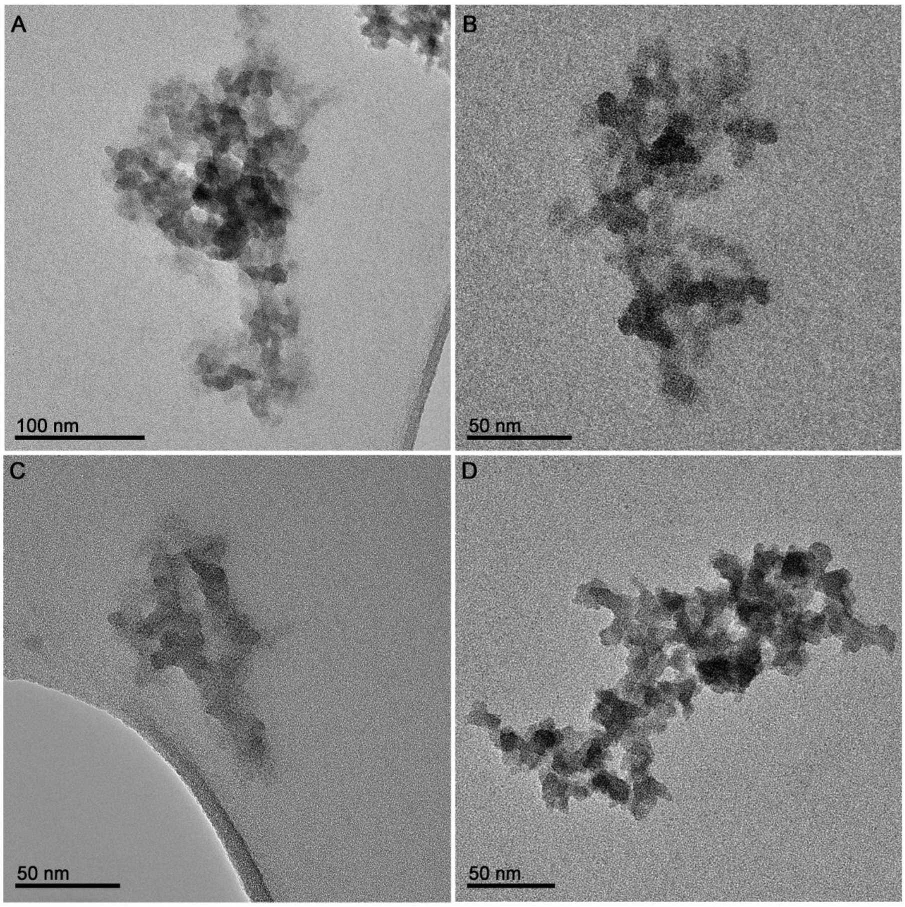

Figure S2. Morphology of C-UiO-66-NH_2 characterized by TEM.

Herein, more images of the TEM morphology of the UiO-66-NH_2 are provided (**Figure S2**). Since UiO-66-NH_2 particles are very sensitive to the electron beam and they melt when scanned under a transmission electron microscope, it is difficult to obtain a very clear image of C-UiO-66-NH_2 under high magnification. However, based on TEM images, very small particles were still be observed, with diameters of ~10 nm.

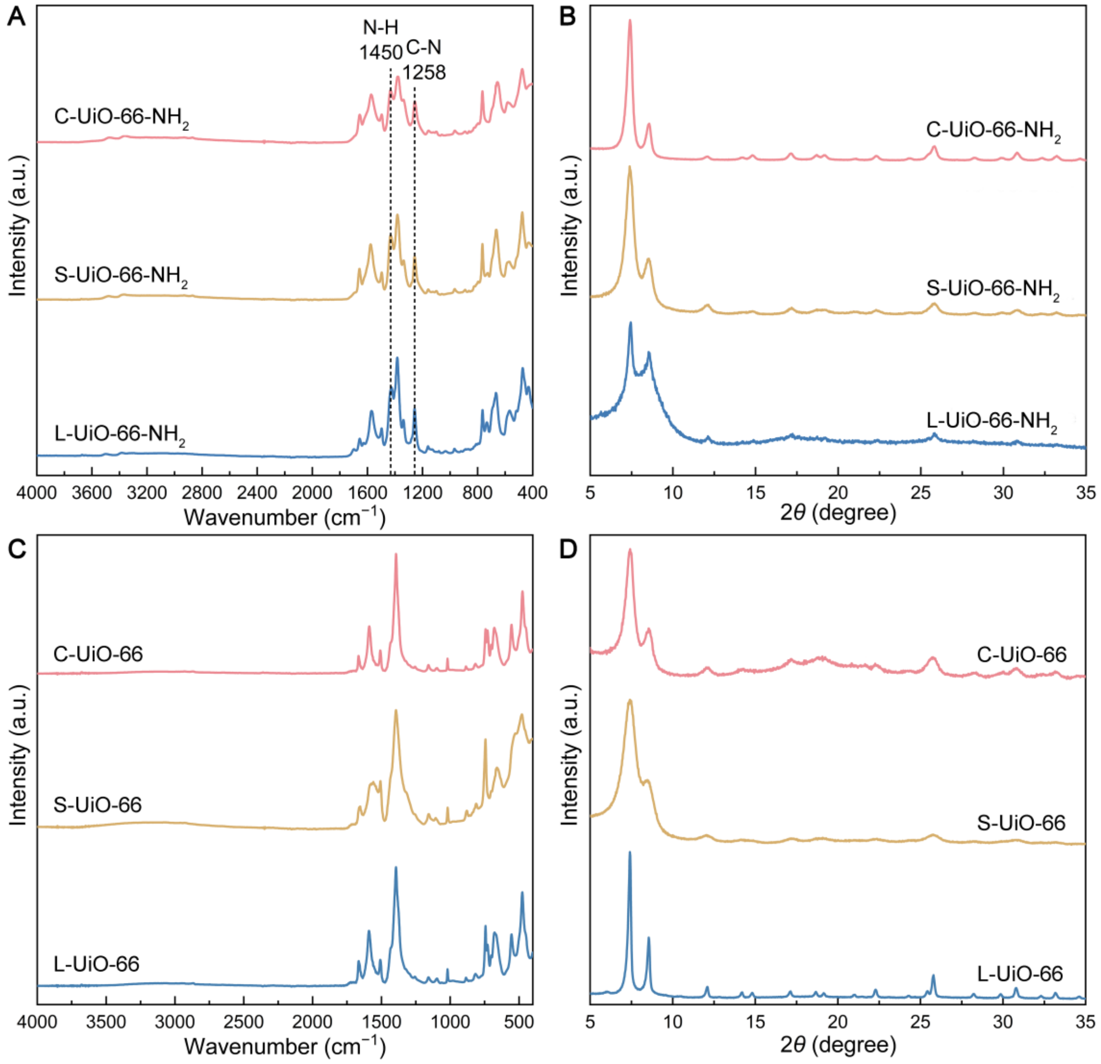

Figure S3. FTIR (A) and XRD (B) spectra of L-UiO-66-NH_2, S-UiO-66-NH_2, and C-UiO-66-NH_2; FTIR (C) and XRD (D) spectra of L-UiO-66, S-UiO-66, and C-UiO-66.

According to **Figure S3**, FTIR and XRD prove the structural consistency among the L-UiO-66-NH_2, S-UiO-66-NH_2, and C-UiO-66-NH_2, as well as among L-UiO-66, S-UiO-66, and C-UiO-66 according to the positional correspondence of the peaks.

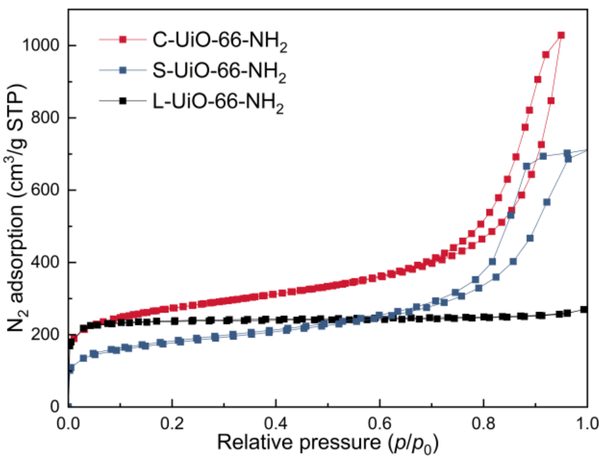

Figure S4. Nitrogen (N_2) adsorption/desorption isotherms of C-UiO-66-NH_2, S-UiO-66-NH_2, and L-UiO-66-NH_2.

The Brunauer-Emmett-Teller (BET) surface area of C-UiO-66-NH_2, S-UiO-66-NH_2, and L-UiO-66-NH_2 is 895 m^2/g, 642 m^2/g, and 928 m^2/g, respectively, based on nitrogen (N_2) adsorption isotherms (**Figure S4**). The similar BET surface area between the C-UiO-66-NH_2 and L-UiO-66-NH_2 demonstrates that the C-UiO-66-NH_2 shows the same frame structure as the L-UiO-66-NH_2. Besides, CQD modulation may increase the orderliness of the crystal framework by reducing the dislocations that occur in secondary nucleation, so that C-UiO-66-NH_2 has a higher specific surface area than S-UiO-66-NH_2.

Both the C-UiO-66-NH_2 and S-UiO-66-NH_2 show Type IV isotherms, which should be attributed to the mesoporosity of the powder due to the presence of voids between the small particles [3].

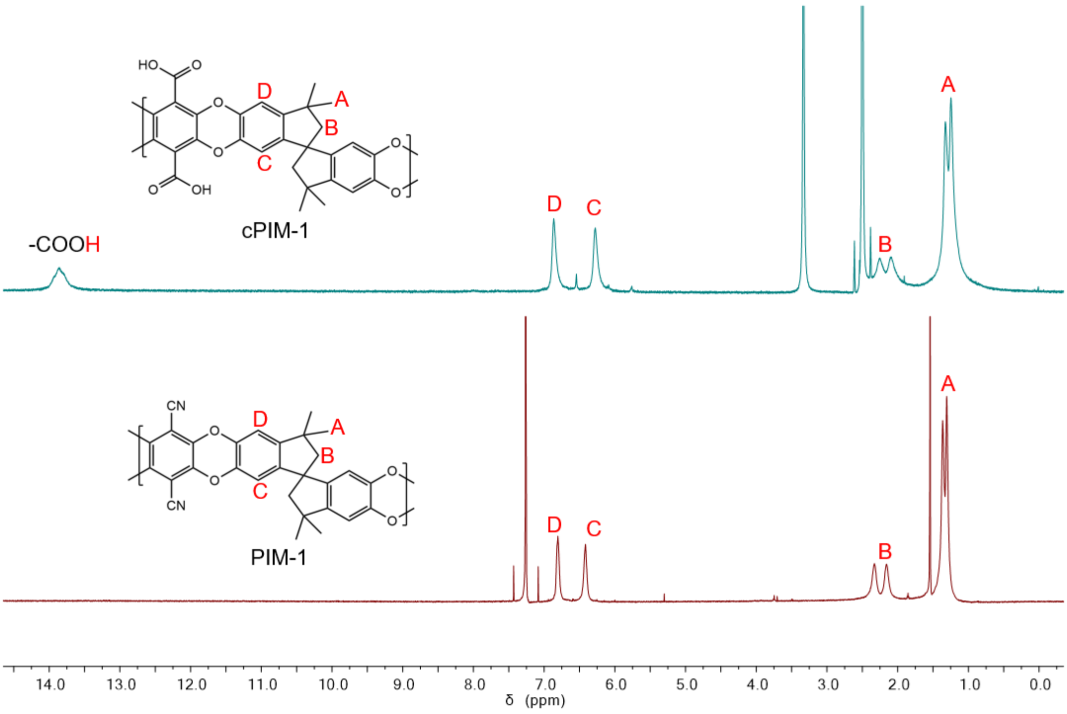

Figure S5. Proton nuclear magnetic resonance (^{1}H NMR) spectra of cPIM-1 and PIM-1, insets are the chemical structures, and proton assignments are labelled on the chemical structures.

The ^{1}H NMR spectra for PIM-1 and cPIM-1 (**Figure S5**) demonstrates a clear distinction between these two polymers, where a small broad signal at around 13–14 ppm designates the –COOH proton.

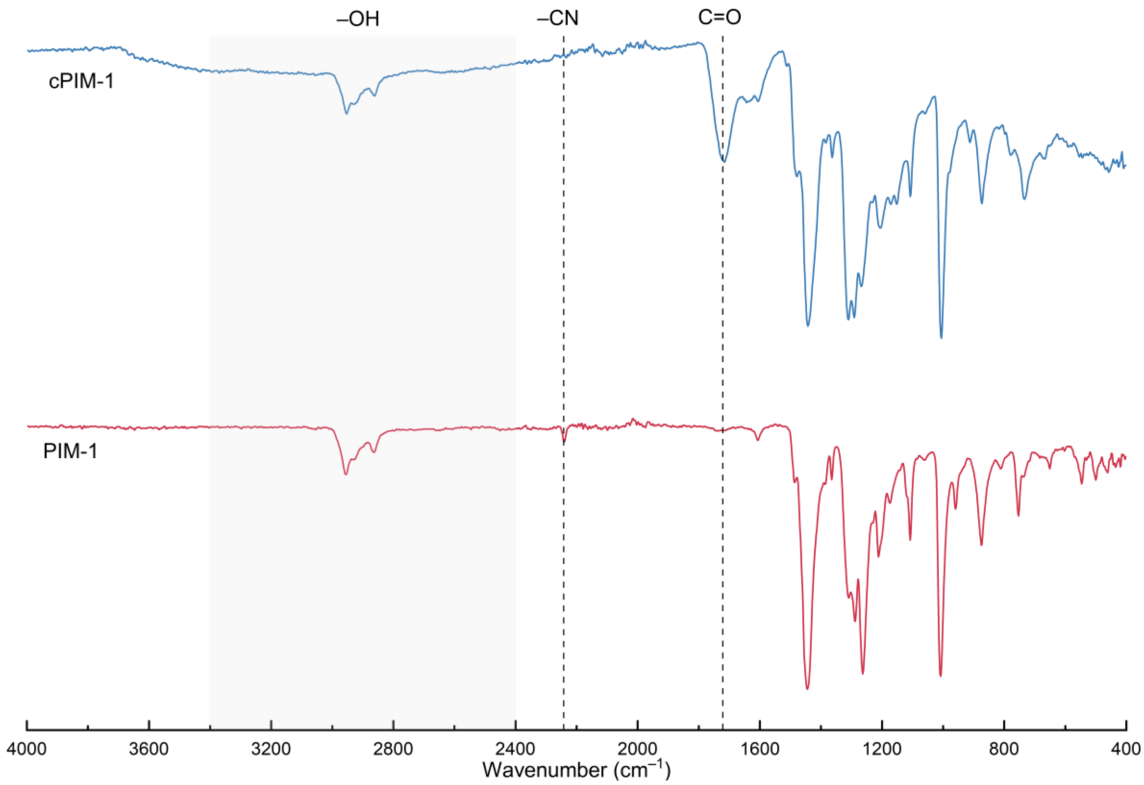

Figure S6. FTIR spectra of PIM-1 and cPIM-1. Bands associated with carboxylic acid and nitrile moieties are shaded, respectively.

As shown in **Figure S6**, after acid hydrolysis, the characteristic peak of the –CN stretching vibrations at 2239 cm^{-1} in PIM-1 diminished and converted to the characteristic peaks of –COOH at 1715 cm^{-1} and a broad peak around 2400–3400 cm^{-1}, which correspond to the C=O and O–H stretching vibrations, respectively.

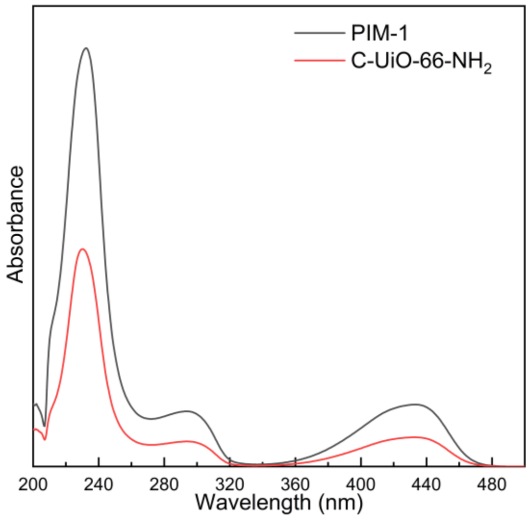

Figure S7. UV-Vis absorbance spectra of the PIM-1 in THF solution and the supernatant of the mixture of C-UiO-66-NH_2 with PIM-1 in THF.

To estimate the PIM-1 loading on the C-UiO-66-NH_2, supernatants from the mixture of PIM-1 and C-UiO-66-NH_2 were analysed by UV-Vis spectroscopy (to determine the unused PIM-1 contained in them) (**Figure S7**). The PIM-1 (solubilized in THF) shows the characteristic absorbance band at 232 nm. The loading of cPIM-1 on C-UiO-66-NH_2 was 243.0 mg/g.

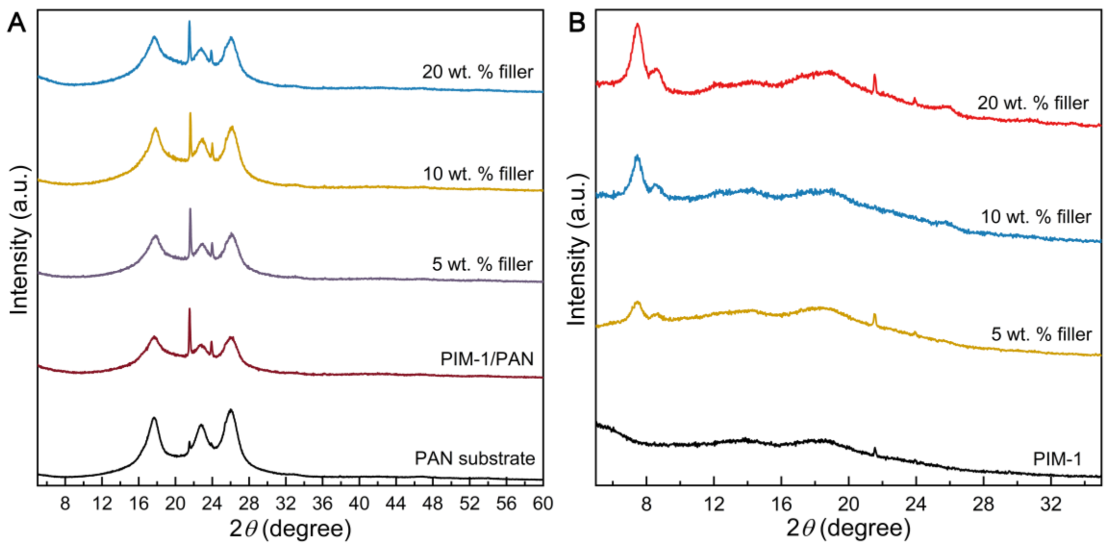

Figure S8. XRD spectra of PIM-1 TFN (A) and thick film (B) with different loadings of cPIM-1/C-UiO-66-NH_2.

XRD has a typical penetration depth of around 10~20 μm. As the PIM-1 active layer is only ~2 μm, most of the signals detected are from the PAN substrate underneath the PIM-1 active layer, therefore, the signals from the PIM-1 layer (or PIM-1 with fillers) can hardly be differentiated from the XRD spectra of the TFN membranes (**Figure S8A**). However, in the XRD of the thick film (~50 μm), with the higher thickness of the membrane, the signals from the PIM-1 (or PIM-1 with fillers) can be detected, and a peak corresponding to filler can be found. The intensity of the peak becomes stronger with increasing loading (**Figure S8B**), demonstrating the successful incorporation of the filler.

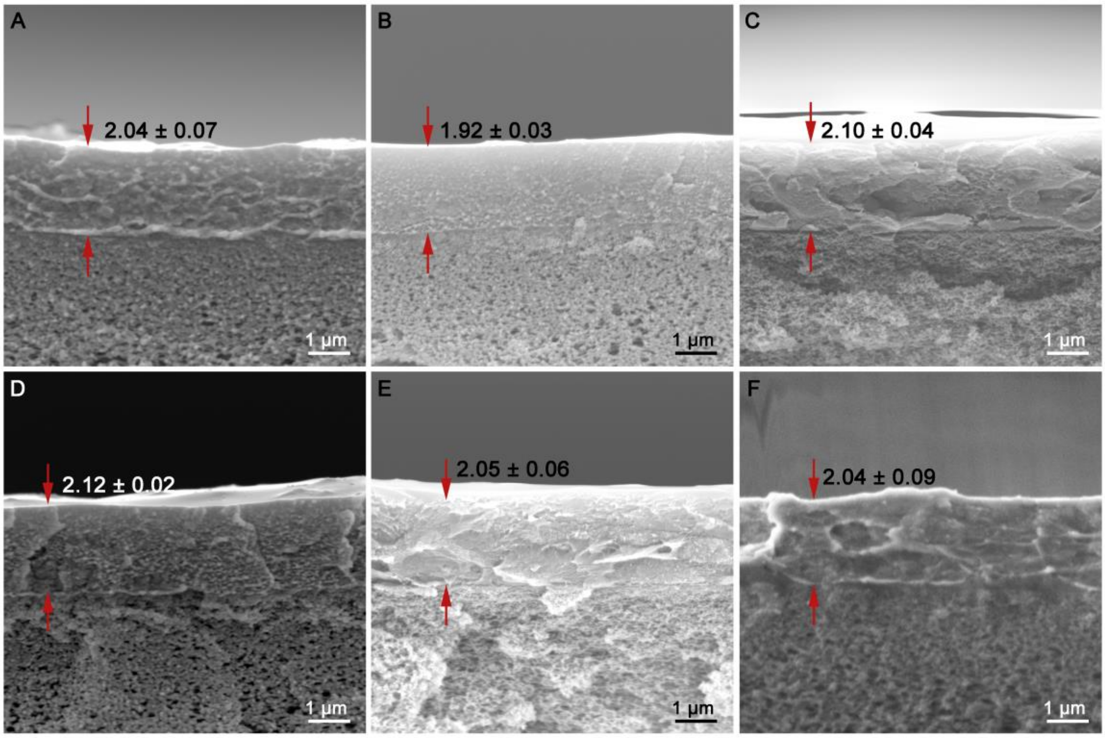

Figure S9. SEM of the cross-section of the PIM-1 TFN with different loadings of cPIM-1/C-UiO-66-NH_2 (A. 0 wt. %; B: 5.0 wt. %; C: 6.5 wt. %; D: 7.5 wt. %; E: 8.5 wt. %; F: 10 wt. %).

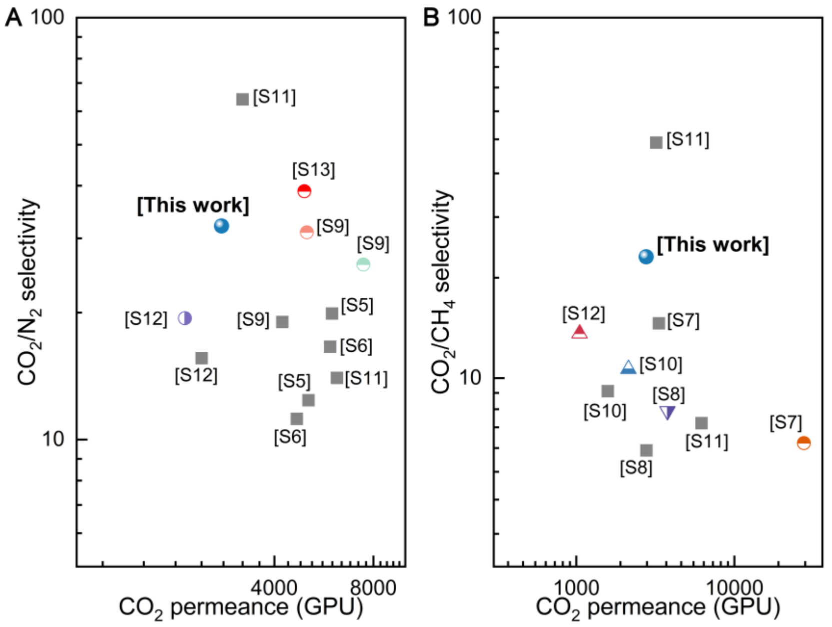

Figure S10. Selectivity versus permeability plots for typical gas pairs (A) CO_2/N_2 and (B) CO_2/CH_4 of PIM-1 TFCs and TFNs fresh membranes (grey squares refer to pure PIM-1 (or pure modified PIM-1) TFCs, circle and triangles refer to PIM-1 TFNs with different filler. See SI for full data set, Table S4).

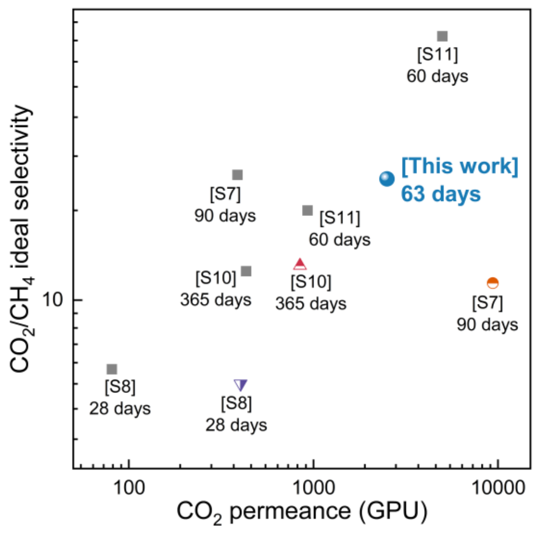

Figure S11. Ideal selectivity versus permeability plots for CO_2/CH_4 of aged PIM-1 TFCs and TFNs membranes (grey squares refer to pure PIM-1 (or pure modified PIM-1) TFCs, circle and triangles refer to PIM-1 TFNs with different fillers. See SI for full data set, Table S4).

Table S1. Elemental analysis of PIM-1 and cPIM-1, and hydrolysis conversion calculations.

Polymer	C (%)	H (%)	N (%)	N/C ratio	Conversion (%)
PIM-1	74.4	4.3	6.0	0.08	0
cPIM-1	66.3	4.4	0.4	0.0059	93

To confirm the level of hydrolysis, elemental analysis was conducted for the PIM-1 and the cPIM-1. The weight percentage of C, H, and N was listed in **Table S1**. The hydrolysis conversion was calculated according to **Eq. (S4)**.

$$Conversion = \frac{\left(N/C\ ratio\right)_{PIM-1} - \left(N/C\ ratio\right)_{cPIM-1}}{\left(N/C\ ratio\right)_{PIM-1}} \tag{S4}$$

Based on the conversion rate, 93% of –CN groups were converted to –COOH groups, demonstrating the successful synthesis of the cPIM-1.

Table S2. Mixed gas permeation properties of PIM-1 and PIM-1 TFN membranes with different filler loadings. Permeation data is calculated based on the average of at least three measurements, errors represent the standard errors.

Filler	Aging days	K_{CO2} (GPU)	K_{N2} (GPU)	$\alpha_{CO2/N2}$
No filler	1	3140.1±421.3	241.0±44.2	13.3±1.8
	4	1751.7±326.3	110.5±38.6	16.7±2.6
	7	1031.9±64.0	71.9±7.9	14.3±3.3
cPIM-1/C-UiO-66-NH_2 (7.5 wt. %)	1	1725.1±389.2	92.7±27.2	18.7±2.6
	4	1755.3±309.1	87.8±20.3	19.5±2.9
	7	1712.0±260.6	86.0±17.9	21.1±2.2
cPIM-1/C-UiO-66-NH_2 (8.5 wt. %)	1	1627.3±222.8	90.7±14.3	20.5±1.3
	4	1692.5±269.3	82.0±17.5	21.2±1.2
	7	1688.0±151.1	83.5±11.0	21.5±0.6

Table S3. Single gas permeation properties of PIM-1 and PIM-1 TFN membranes with different fillers and filler loadings. Permeation data is calculated based on the average of at least three measurements, errors represent the standard errors.

Filler	Aging days	K_{CO2} (GPU)	Normalised P_{CO2}[a]	K_{CH4} (GPU)	K_{N2} (GPU)	$\alpha_{CO2/CH4}$	$\alpha_{CO2/N2}$
No filler	1	4599±478	1	424±5	219.3±0.4	13.0±2.5	21.0±2.3
	7	2161±310	0.47	117±54	64±9	17.4±2.8	23.8±4.8
	28	1331±76	0.29	34±10	72.3±2	12.1±1.8	15.4±2.8
cPIM-1/C-UiO-66-NH_2 (5.0 wt. %)	1	3355±655	1	245±101	131±24	16.4±1.4	27.3±3.0
	7	2743±32	0.74	163±13	84±5	17.4±2.1	28.3±1.8
	28	2493±231	0.68	153±22	89±11	12.9±0.3	22.5±0.3
cPIM-1/C-UiO-66-NH_2 (6.5 wt. %)	1	2736±267	1	129±70	86±29	16.5±1.8	27.4±2.2
	7	2882±14	1.05	148±2	87±7	18.9±3.5	28.5±3.7
	28	2389±174	0.87	135±38	83±16	18.0±1.2	28.8±0.7
cPIM-1/C-UiO-66-NH_2 (7.5 wt. %)	1	2763±29	1	127±3	86±17	18.1±2.7	29.4±1.9
	7	2777±161	1.00	124±33	86±33	18.1±2.6	31.5±7.8
	28	2632±149	0.95	129±8	81±29	20.3±3.3	28.7±1.1
	63	2523±93	0.91	129±26	68±3	20.3±3.4	37.1±0.4
cPIM-1/C-UiO-66-NH_2 (8.5 wt. %)	1	2664±392	1	143±13	87±7	19.0±1.1	29.2±2.6
	7	2597±53	0.97	131±34	73±14	18.8±4.2	31.6±5.8
	28	2559±265	0.96	123±35	75±3	20.6±4.2	33.9±2.3
	63	2504±124	0.94	109±8	67±12	23.8±2.1	37.2±4.2
cPIM-1/C-UiO-66-NH_2 (10.0 wt. %)	1	2439±345	1	152±42	83±22	14.8±1.0	22.3±1.5
	7	2265±6	0.92	128±5	74±4	19.7±3.3	31.3±2.6
	28	2587±68	0.93	107±8	72±3	21.4±2.5	31.7±2.1

cPIM-1/C-UiO-66 (8.5 wt. %)	1	2564±108	1	212±69	124±41	12.1±3.7	20.7±2.0
	7	1629±272	0.63	96±12	62±17	19.3±2.3	30.9±1.0
	28	1194±126	0.47	94.5±0.0	83±3	14.4±0.1	16.4±0.7
C-UiO-66-NH_2 (8.5 wt. %)	1	2620±250	1	186±35	99±22	14.4±3.3	27.5±4.1
	7	1881±363	0.72	119±32	75±16	16.6±3.8	28.9±2.4
	28	1362±6	0.52	104±12	64±10	16.7±4.3	18.3±5.5
cPIM-1/S-UiO-66-NH_2 (8.5 wt. %)	1	3441±132	1	190±26	132±16	18.1±2.4	26.1±3.1
	7	1735±159	0.50	95±10	72±19	18.3±1.2	24.2±1.8
	28	1337±92	0.39	70±24	56±8	19.4±2.3	23.9±0.4
cPIM-1/L-UiO-66-NH_2 (8.5 wt. %)	1	5038±398	1	294±23	216±11	17.1±1.2	23.3±4.1
	7	2352±363	0.47	155±42	103±31	16.4±1.5	22.9±3.5
	28	1649±140	0.33	96±17	75±9	17.3±2.8	22.0±2.1

[a] Normalised P_{CO2} after aging is calculated based on the CO_2 permeance on Day 1.

Table S4. Overview of PIM-1 TFN membranes for CO_2/N_2 and CO_2/CH_4 separation. Permeation data in this work is calculated based on the average of at least three measurements, errors represent the standard errors.

Literature	Membranes	Aging days	K_{CO2} (GPU)	Normalised $K_{CO2}{}^{a}$	K_{CH4} (GPU)	K_{N2} (GPU)	$\alpha_{CO2/CH4}$	$\alpha_{CO2/N2}$
[5]	PIM-1, #1, cyclic	1	5079	1	-	409	-	12.4
		7	1207	0.24	-	89	-	13.6
		28	295	0.06	-	61	-	6.5
	PIM-1, # 3b, high network content (7.8 wt. %)	1	5985	1	-	303	-	19.9
		7	1648	0.28	-	82	-	20
		28	493	0.08	-	35	-	14.1
[6]	PIM-1, #1, network content 0.8 wt. %	1	4678±518	1	-	419±61	-	11.2
		7	1114±144	0.24	-	96±1	-	11.7
		28	134±28	0.03	-	37.6±28	-	3.6
	PIM-1, #3, network content 85.3 wt. %	1	1514±333	1	-	804±475	-	1.9
		7	619±193	0.41	-	817±266	-	0.8
		28	1377±292	0.91	-	1786±517	-	0.8
	Blended PIM-1, 20% #3 in #1	1	5910±704	1	-	357±49	-	16.6
		7	3129±848	0.53	-	183±49	-	17.1
		28	3480±209	0.59	-	233±6	-	14.9
		130	34.9±10	0.01	-	3.3±1.6	-	10.7
[7]	PIM-1/C-HCP (6 wt. %)	1	27530	1	4197	-	6.6	-
		7	15352	0.56	1652	-	9.3	-
		90	9379	0.34	834	-	11.3	-
	PIM-1	1	3331	1	234	-	14.2	-

		7	1441	0.43	77	-	18.6	-
		90	388	0.12	-	-	24.5	-
[8]	PIM-1/S-SN0.05	0	3771±57	1	433±69	-	8.1±7.2	-
		7	1387	0.37	143	-	9.6	-
		28	403±43	0.11	73±17	-	5.5±1.1	-
	PIM-1	0	2778±1010	1	441±108	-	6.3±1.1	-
		7	201	0.07	32	-	6.3	-
		28	81±10	0.03	13±3	-	6.1±0.3	-
[9]	PIM-1	1	4230	1	-	-	-	19
		21	490	0.12	-	-	-	31
	PIM-1/MOF-74-Ni	1	5020	1	-	-	-	31
		7	3240	0.65	-	-	-	-
		56	1200	0.24	-	-	-	30
	PIM-1/UiO-66-NH_2	1	7460	1	-	-	-	26
		28	900	0.12	-	-	-	28
[10]	PIM-1	1	1583±400	1	190±78	-	9.2±2.0	-
		66	669	0.42	29	-	23.4	-
		365	432±4	0.27	35±2	-	12.3±0.7	-
	1.0rHGO-TAPA	1	1050±70	1	79±7	-	13.3±0.3	-
		365	846±37	0.81	67±7	-	12.8±2.0	-
[11]	Linear PIM-1	1	6200±720	1	830±98	430±46	7.5±0.6	14±0.8
		6	3000±1200	0.48	320±210	180±110	12±5.9	20±7.8
		26	1800±1300	0.29	160±170	94±93	17±8.3	23±6.8
		60	930±400	0.15	52±25	39±13	19±1.9	23±4.3
	Branched-cPIM-1-73&81%	1	3200±700	1	83±48	58±31	45±16	64±26

		6	3100±790	0.97	69±33	50±16	49±15	63±4.8
		20	3000±970	0.94	66±37	47±23	55±29	76±47
		60	5000±670	1.56	88±41	55±28	66±39	110±69
[4]	PIM-1 stored in methanol (D1)	1	1000±170	1	-	-	20±3.6	-
		7	1100±533	1.10	-	-	14±0.7	-
		28	2200±400	2.20	-	-	8.8±1.1	-
	PIM-1 stored in methanol (B1 3%)	1	2100±6	1	-	-	12±3.7	-
		7	2800±170	1.33	-	-	6.2±0.4	-
		28	3400±95	1.62	-	-	5.6±0.4	-
[12]	PIM-1/OAPS (5 wt. %)	1	2138	1	201.2	110.4	10.6	19.4
		30	320.7	0.15	-	-	-	26.3
	PIM-1	1	2403	1	275	154.1	8.74	15.6
		30	288.4	0.12	-	-	-	27.5
[13]	PIM-1/PTMSP#3	1	4930	1	-	-	-	38.8
		94	297	0.06	-	-	-	37.1
	PIM-1/PTMSP#1	1	8010	1	-	-	-	35.8
		94	208	0.03	-	-	-	34.6
	PIM-1/PTMSP#6	1	3010	1	-	-	-	55.7
		94	281	0.09	-	-	-	56.2
This work	PIM-1	1	4599±478	1	424±5	219.3±0.4	13.0±2.5	21.0±2.3
		7	2161±310	0.47	117±54	64±9	17.4±2.8	23.8±4.8
		28	1331±76	0.29	34±10	72±2	12.1±1.8	15.4±2.8
	PIM-1/C-UiO-66-NH_2/cPIM-1 (8.5 wt. %)	1	2664±392	1	143±13	87±7	19.0±1.1	29.2±2.6
		7	2597±53	0.97	131±34	73±14	18.8±4.2	31.6±5.8
		28	2559±265	0.96	123±35	75±3	20.6±4.2	33.9±2.3

	63	2504±124	0.94	109±8	67±12	23.8±2.1	37.2±4.2

[a] Normalised P_{CO2} after aging is calculated based on the CO_2 permeance on Day 1.

References:

[1] N. Du, J. Song, G. P. Robertson, I. Pinnau, M. D. Guiver, *Macromol. Rapid Comm.* **2008**, *29*, 783-788.

[2] K. M. Rodriguez, A. X. Wu, Q. Qian, G. Han, S. Lin, F. M. Benedetti, H. Lee, W. S. Chi, C. M. Doherty, Z. P. Smith, *Macromolecules* **2020**, *53*, 6220-6234.

[3] B. Ghalei, K. Sakurai, Y. Kinoshita, K. Wakimoto, A. P. Isfahani, Q. Song, K. Doitomi, S. Furukawa, H. Hirao, H. Kusuda, S. Kitagawa, E. Sivaniah, *Nat. Energy* **2017**, *2*, 17086.

[4] M. Yu, A. B. Foster, C. A. Scholes, S. E. Kentish, P. M. Budd, *ACS Macro Lett.* **2023**, *12*, 113-117.

[5] A. B. Foster, M. Tamaddondar, J. M. Luque-Alled, W. J. Harrison, Z. Li, P. Gorgojo, P. M. Budd, *Macromolecules* **2020**, *53*, 569-583.

[6] A. B. Foster, J. L. Beal, M. Tamaddondar, J. M. Luque-Alled, B. Robertson, M. Mathias, P. Gorgojo, P. M. Budd, *J. Mater. Chem. A* **2021**, *9*, 21807-21823.

[7] R. S. Bhavsar, T. Mitra, D. J. Adams, A. I. Cooper, P. M. Budd, *J. Membr. Sci.* **2018**, *564*, 878-886.

[8] S. Mohsenpour, Z. Guo, F. Almansour, S. M. Holmes, P. M. Budd, P. Gorgojo, *J. Membr. Sci.* **2022**, *661*, 120889.

[9] M. Liu, M. D. Nothling, P. A. Webley, J. Jin, Q. Fu, G. G. Qiao, *Chem. Eng. J.* **2020**, *396*, 125328.

[10] F. Almansour, M. Alberto, A. B. Foster, S. Mohsenpour, P. M. Budd, P. Gorgojo, *J. Mater. Chem. A* **2022**, *10*, 23341-23351.

[11] M. Yu, A. B. Foster, M. Alshurafa, J. M. Luque-Alled, P. Gorgojo, S. E. Kentish, C. A. Scholes, P. M. Budd, *J. Membr. Sci.* **2023**, 121697.

[12] Y. Kinoshita, K. Wakimoto, A. H. Gibbons, A. P. Isfahani, H. Kusuda, E. Sivaniah, B. Ghalei, *J. Membr. Sci.* **2017**, *539*, 178-186.

[13] I. Borisov, D. Bakhtin, J. M. Luque-Alled, A. Rybakova, V. Makarova, A. B. Foster, W. J. Harrison, V. Volkov, V. Polevaya, P. Gorgojo, E. Prestat, P. M. Budd, A. Volkov, *J. Mater. Chem. A* **2019**, *7*, 6417-6430.
